# Casein Kinase II Induced Polymerization of Soluble TDP-43 into Filaments Is Inhibited by Heat Shock Proteins

**DOI:** 10.1371/journal.pone.0090452

**Published:** 2014-03-04

**Authors:** Yari Carlomagno, Yongjie Zhang, Mary Davis, Wen-Lang Lin, Casey Cook, Judy Dunmore, William Tay, Kyle Menkosky, Xiangkun Cao, Leonard Petrucelli, Michael DeTure

**Affiliations:** Department of Neuroscience, Maya Clinic, Jacksonville, Florida, United States of America; Hertie Institute for Clinical Brain Research and German Center for Neurodegenerative Diseases, Germany

## Abstract

**Background:**

Trans-activation Response DNA-binding Protein-43 (TDP-43) lesions are observed in Amyotrophic Lateral Sclerosis (ALS), Frontotemporal Lobar Degeneration with ubiquitin inclusions (FTLD-TDP) and 25–50% of Alzheimer's Disease (AD) cases. These abnormal protein inclusions are composed of either amorphous TDP-43 aggregates or highly ordered filaments. The filamentous TDP-43 accumulations typically contain clean 10–12 nm filaments though wider 18–20 nm coated filaments may be observed. The TDP-43 present within these lesions is phosphorylated, truncated and ubiquitinated, and these modifications appear to be abnormal as they are linked to both a cellular heat shock response and microglial activation. The mechanisms associated with this abnormal TDP-43 accumulation are believed to result in a loss of TDP-43 function, perhaps due to the post-translational modifications or resulting from physical sequestration of the TDP-43. The formation of TDP-43 inclusions involves cellular translocation and conversion of TDP-43 into fibrillogenic forms, but the ability of these accumulations to sequester normal TDP-43 and propagate this behavior between neurons pathologically is mostly inferred. The lack of methodology to produce soluble full length TDP-43 and recapitulate this polymerization into filaments as observed in disease has limited our understanding of these pathogenic cascades.

**Results:**

The protocols described here generate soluble, full-length and untagged TDP-43 allowing for a direct assessment of the impact of various posttranslational modifications on TDP-43 function. We demonstrate that Casein Kinase II (CKII) promotes the polymerization of this soluble TDP-43 into 10 nm diameter filaments that resemble the most common TDP-43 structures observed in disease. Furthermore, these filaments are recognized as abnormal by Heat Shock Proteins (HSPs) which can inhibit TDP-43 polymerization or directly promote TDP-43 filament depolymerization.

**Conclusion:**

These findings demonstrate CKII induces polymerization of soluble TDP-43 into filaments and Hsp90 promotes TDP-43 filament depolymerization. These findings provide rational for potential therapeutic intervention at these points in TDP-43 proteinopathies.

## Introduction

### TDP-43 Protein

**Figure 1:**
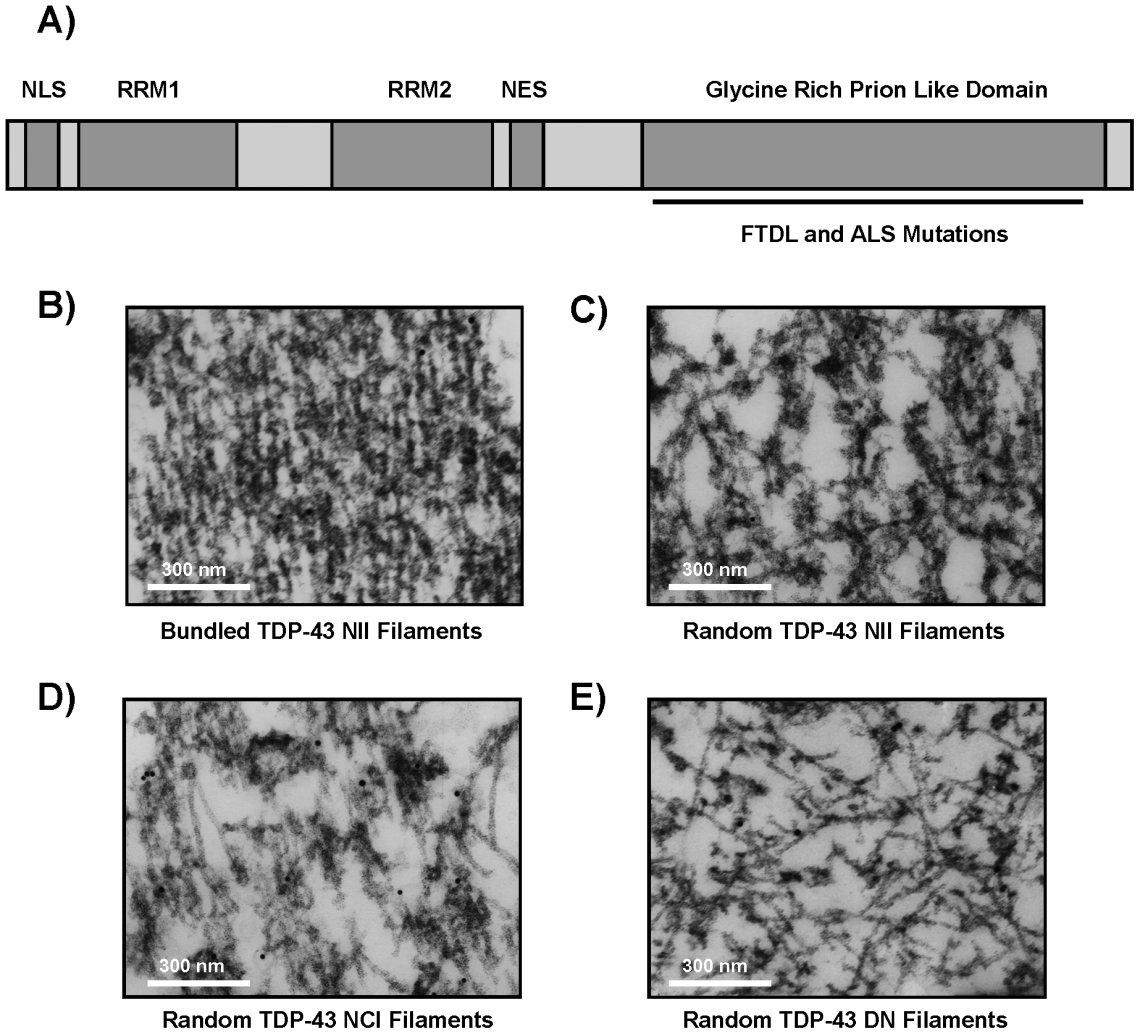
Structure of TDP-43 Protein and FTLD Inclusions. A) Schematic representation of rTDP-43 protein indicates the location of two RNA-recognition motifs (RRM1/2), the nuclear localization signal (NLS), nuclear export signal (NES) and the glycine rich, prion like domain containing FTLD and ALS mutations. Sections from FTLD-TDP cases display neuronal intranuclear inclusions composed of B) clean 10–12 nm TDP-43 filaments that can laterally associate into bundles and also C) granular TDP filaments that are not associated laterally and appear wider at 18–20 nm due to the association of this granular material with the 10–12 nm filaments. This randomly oriented distribution of uncoated 10–12 nm TDP-43 filaments is also observed in D) neuronal cytoplasmic inclusions and E) dystrophic neurites of FTLD-TDP cases. The 10 nm gold particles in these FTLD panels represent anti-TDP-43 labeling.

Trans-activation response DNA-binding protein (TDP-43) is highly conserved among species and ubiquitously expressed in humans [1]. This 43 kDa protein is encoded by the TARDBP gene on chromosome 1, and TARDBP mutations have been genetically linked to ubiquitin-positive, Tau-negative inclusions in Amyotrophic Lateral Sclerosis (ALS) and subtypes of Frontotemporal Lobar Degeneration (FTLD) linked to TDP-43 (FTLD-TDP) and motor neuron disease (FTLD-MND) [2]. As shown in **Figure 1A**, TDP-43 contains two RNA recognition motifs that are involved in its function in RNA stabilization and processing, while the carboxy-terminus is believed to drive a toxic gain of function, as the majority of ALS and FTLD-TDP-linked mutations are found in this glycine-rich region of the protein [3], [4], [5], [6]. This carboxy-terminal portion of the TDP-43 molecule shares homology with members of the heterogeneous nuclear ribonucleoprotein (hnRNP) family and can bind hnRNP A/B and hnRNP A1 [7]. In addition, the C-terminus appears to contain prion-like sequences that can promote aggregation and stress granule formation, potentially reducing its normal RNA functional activities [8], [9], [10], [11]. TDP-43 contains both nuclear import and export sequences, and is typically observed in the nucleus where it co-localizes with nuclear substructures like Cajal bodies, the Gemini of coiled bodies and foci associated with promyelocytic leukemia or spliceosomal protein SC35, suggesting a role in RNA transcription, stabilization and splicing [12]. Evidence indicates that FTLD-TDP can be caused by progranulin mutations that lead to cytoplasmic aggregates of TDP-43, while ALS can be caused by TARDBP mutations and is associated with perinuclear TDP-43 aggregates [13], [14]. These findings, coupled with the observation that many TDP-43 mutations occur around the prion-like motif, suggest that ALS and FTLD-TDP may be caused by aggregation of TDP-43 which inhibits its normal cellular activities. This loss of TDP-43 function may ultimately be instrumental in disease pathogenesis, and additionally the TDP-43 aggregates may sequester other essential RNAs and proteins [15]. Furthermore, the tremendous variety of reported TDP-43 inclusions suggests distinct mechanisms that may result from the misregulation of disease-specific and potentially overlapping signaling pathways that ultimately lead to TDP-43 accumulation and the subsequent functional deficits characteristic of all diseases with TDP-43 inclusions [16], [17].

### TDP-43 Protein in Disease

Since the identification of TDP-43 in neuronal cytoplasmic inclusions (NCI) and dystrophic neurites (DN) in FTLD and neuronal intranuclear inclusions (NII), skein-like inclusions and glial inclusions in ALS, it has become apparent that abnormal TDP-43 localization and accumulation into amorphous aggregates and filamentous structures must be related to numerous pathological stressors [18], and this has been further exemplified by the identification of TDP-43 accumulation as a secondary pathology in Alzheimer's disease (AD), Parkinson's disease (PD), and Huntington's disease cases [19], [20]. Ultrastructural analysis of normal nuclear TDP-43 in control cases using electron microscopy (EM) suggests that TDP-43 is not typically observed in filamentous form; and though some disease-associated TDP-43 inclusions appear to be unstructured, many are characterized by filamentous TDP-43 inclusions containing a variety of structures [21]. In FTLD-TDP, it is reported that neuronal cytoplasmic inclusions may contain granular TDP-43 or loosely associated clean TDP filaments ranging in width from 4–16 nm with an average diameter of 10–12 nm (**Figure 1D**) which are correlated with a subsequent decrease in nuclear TDP-43. These TDP-43 filaments are similar in dimension to those observed in dystrophic neurites in FTLD-TDP (**Figure 1E**), but there the TDP-43 filaments can also be densely packed into parallel arrays, in addition to the more granular aggregates which still contain some filaments in these dystrophic neurites. Similar variation is observed in the structural composition of neuronal intranuclear inclusions in FTLD-TDP, with both loosely associated or densely packed TDP-43 filaments (**Figure 1B**), which can range in width from 9–50 nm, with an average diameter of 18–20 nm for the granular filamentous aggregates (**Figure 1C**), in addition to the more commonly observed 10–12 nm filaments [21]. Furthermore, this variety of filamentous structures that can form from TDP-43 is not limited to FTLD-TDP as randomly oriented and bundled TDP-43 filaments have been reported for AD, Pick's disease and Lewy body disease cases as well, and immuno-gold labeling of these sections with phosphorylation-specific TDP-43 and ubiquitin antibodies confirms these filaments also may contain post-translationally modified TDP-43 [16], [21], [22].

### Assembly Properties of TDP-43 Protein

The observation abnormal nuclear and cytoplasmic TDP-43 aggregation can correlate with disease progression in ALS and FTLD indicates that if this accumulation can be inhibited the resulting phenotypic changes may be alleviated magnifying the importance of understanding the mechanisms leading to these TDP-43 lesions [15], [23]. Initial reports indicated that Casein Kinase I (CKI) and II (CKII) were each capable of phosphorylating partially purified recombinant TDP-43 (rTDP-43) at the S379, S403/404 and S409/410 sites implicated in FTLD cases [16], and the CKI-induced a large retardation of TDP-43 mobility that correlated with TDP-43 oligomer and filament assembly that was not observed with CKII or glycogen synthase kinase – 3β (GSK-3β). Interpretation of these results was limited due to the impurity of this rTDP-43, and overall studies on the assembly properties of TDP-43 have been hindered by the extreme insolubility when TDP-43 is bacterially-expressed and purified to homogeneity. Specifically soluble rTDP-43 begins to aggregate in a carboxy-terminus dependent manner within five minutes of being clarified once agitation is applied [24]. This aggregation was accelerated by the presence of the G294A, Q331K or M337V mutations; and although these were predominantly amorphous structures for WT TDP-43, some 18–20 nm wide granular filaments were observed with longer incubation times [24]. More recently, techniques involving guanidine hydrochloride solubilization of the inclusion bodies formed with rTDP-43 expression have demonstrated that the protein can be refolded upon dilution with a glycerol buffer and can then specifically bind TG repeated DNA[25]. This rTDP-43 is maximally obtained at 2 µM, but it can assemble into weakly thioflavin T (ThT)-positive filaments with agitation, and seed ThT-positive rTDP-43 aggregation *in vitro* and ubiquitin-positive, sarksoyl-insoluble TDP-43 aggregation *in vivo* [25]. This study also demonstrated these atypical 2.6 nm wide rTDP43 filaments contain a pronase resistant core consisting predominantly of the last 100 amino acids of TDP-43, and this filament core has been further refined using synthetic peptides from 286–331 of TDP-43 that share a high homology with the prion protein sequences. These synthetic 46 amino acid peptides form ThT-positive filaments when incubated at 250 µM concentrations, and the 7.7 nm wide filaments are toxic to primary neuronal cultures [10]. In these studies, it was not determined whether TDP-43 peptide filaments were accompanied by sequestration of endogenous TDP-43 as observed with the full-length TDP-43 filaments [10], [25]. To date there is not a published report describing how to generate the 10–12 nm wide TDP-43 filaments most commonly observed in disease tissue nor are there reliable protocols describing how to express and purify soluble untagged wild type rTDP-43 for assembly studies.

### Purposes of the Present Studies

TDP-43 forms amorphous aggregates when isolated but can assemble into filamentous structures when prompted by an activating or nucleating event indicating that endogenous TDP-43 solubility is likely regulated by its binding partners and that when not sequestered it is vulnerable to either aggregation or polymerization [11], [26], [27], [28]. Similar to Tau in AD and α-synuclein in PD, TDP-43 becomes phosphorylated, truncated and ubiquitinated during disease progression until it reaches its final intractable state, stressing the cells, mislocalized and malfunctioning, and the neurons die [29], [30], [31]. Similarly, it is quite reasonable to expect that phosphorylation not only regulates normal TDP-43 functional activities including cellular localization and solubility, but that these pathways may be vulnerable to aberrant signaling pathways, resulting in abnormal modifications and ultimately cell death. In fact, if this were the case, it might also be expected that this abnormal signaling has been anticipated by the cell such that consensus motifs for heat shock protein (HSP) recognition of TDP-43 might be exposed during the pathogenic cascade as occurs with Tau [11], [32], [33]. In tauopathy models recapitulating neurofibrillary tangle (NFT) formation, the accumulation of insoluble non-functioning Tau induces the increased expression of chaperones like Hsp40 and Hsp70, and manipulation of Hsp70 and Hsp90 can promote the degradation of abnormal Tau [34]. Similarly Hsp70 expression is increased in neuroblastoma cells that form ubiquitin and phosphorylation immunoreactive cytoplasmic inclusions that are driven by the expression of caspase-cleaved, carboxy-terminal fragments of TDP-43 [31], [35]. Other chaperones such as Hsp40, Hsc70 and Hsp105 have been identified as binding partners in transfected cells, and Hsp70 and Hsp90 are reported to be central in the degradation of normal and insoluble TDP-43 [31], [36], [37]. Given that phosphorylation alone can promote Tau filament assembly and heat shock proteins recognize it as abnormal and can inhibit Tau polymerization, it was expected that phosphorylation could promote the polymerization of soluble full-length, wild-type rTDP-43 into filaments characteristic of disease and that the heat shock proteins might attenuate or ameliorate this process [38], [39], [40]. These studies attempted to determine if this hypothesis was true, and the results indicate 1) that soluble, full-length, untagged rTDP-43 may be purified to homogeneity using it propensity to aggregate and ammonium sulfate, 2) that kinases implicated in Alzheimer's disease can promote this rTDP-43 polymerization into 10–12 nm wide filaments as observed in FTLD-TDP and related disease and 3) that Hsp70 and 90 can inhibit this TDP-43 filament assembly and even depolymerize assembled TDP-43 filaments indicating they are recognized as abnormal.

## Results

### Purification of Soluble Untagged TDP-43

**Figure 2:**
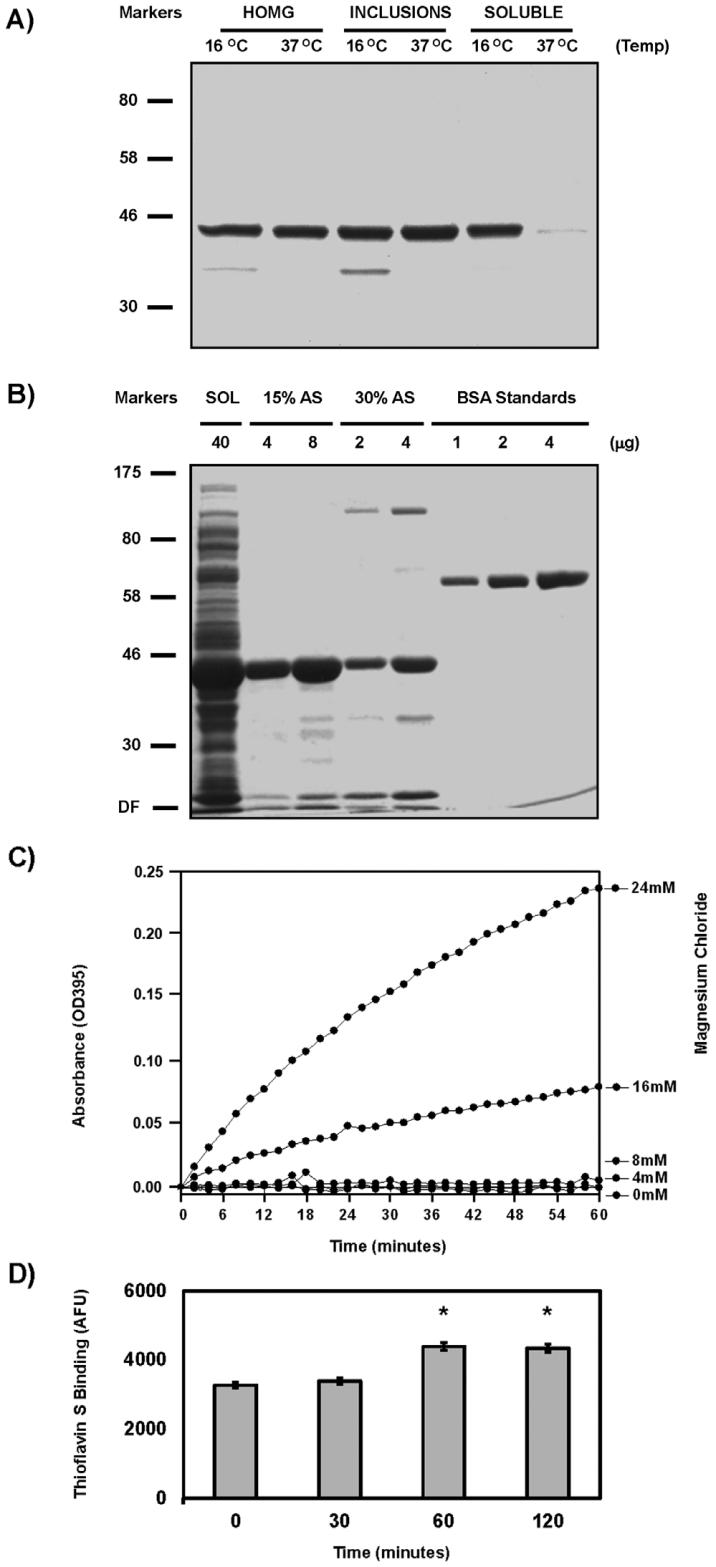
Purity of Soluble rTDP-43. A) Coomassie staining demonstrates that lowering the induction temperature from 37°C to 16°C increased TDP-43 partitioning into the soluble fraction (SOL). B) This soluble rTDP-43 was readily precipitated by both 15% and 30% ammonium sulfate (AS) cuts, but the 15% cut gave better purity and an improved yield. Bovine serum albumin (BSA) was used as standards, and the dye front is marked DF. C) Immediately after clarification, 2.5 µM rTDP-43 was incubated at 37°C and the OD395 was monitored. These reactions contained MgCl_2_ to confirm reports that these cations promote aggregation. D) The Thioflavin S fluorescence increased 1.3 +/− 0.1 fold and was significant at p<0.01 (*).

Previous reports on the *in vitro* assembly of tagged, full-length, wild-type TDP-43 demonstrated that the protein is extremely aggregation-prone when purified to homogeneity [24], [25]. The first report demonstrated histidine-tagged TDP-43 could be expressed bacterially and isolated from the soluble pool after sonication using 10% glycerol to stabilize the proteins, and these materials would aggregate as assessed by low speed centrifugation into filament-like structures, threads and some granular filamentous structures 18-20 nm in width. The key step was filtering the sample using a 0.22 µm cut off to remove particulate material larger than the 800 kDa granular oligomers observed by size exclusion chromatography, but the proportion of 18 nm wide filaments in these samples appeared to be very low compared to the amorphous aggregates based on EM analysis [24]. Subsequently, TDP-43 was isolated from bacterially-expressed inclusion bodies in the insoluble inclusion body fraction, denatured and solubilized by guanidine hydrochloride, before purification again using the histidine tag. The isolates were refolded by flash dilution and clarified by high-speed centrifugation, and the resulting soluble TDP-43 was demonstrated to specifically bind TG repeated DNA and aggregate upon agitation forming sarkosyl-insoluble structures. This material was slightly ThT-positive and appeared to contain thin fibers that coalesced into bundles, but the ThT signal was not robust in comparison to amyloid filaments, and the thin fibers with a width below 3.0 nm were smaller than filaments observed in fixed patient samples [25]. These data however did indicate that it might be possible to express and purify soluble, untagged full-length TDP-43 that was fibrillogenic. To explore this idea, full-length, wild-type TDP-43 expression was induced at 16°C overnight using progressively lower IPTG concentrations to slow the expression and increase the amount of TDP-43 in the soluble partition as shown **in** **Figure 2A**. This material was then isolated to homogeneity using only 15% saturated ammonium sulfate to precipitate the TDP-43, and the salt was then removed by dialysis before high-speed clarification. SDS-PAGE analysis of this material with Coomassie brilliant blue staining demonstrated soluble, full-length, untagged TDP-43 can be isolated by taking advantage of its intrinsic propensity to aggregate, as shown in **Figure 2B**, and this material is over 95% pure based on densitometry calculations of the gels. Purified recombinant soluble TDP-43 was found to be extremely aggregation-prone as reported, and incubation of this soluble TDP-43 at 37°C with agitation resulted in an increase in the turbidity that was dependent on MgCl_2_ as reported, but this led to negligible Thioflavin S fluorescence characteristic of cross-β amyloid structures as shown in **Figure 2C-D** [24], [25]. In fact, this modest 1.3 +/− 0.1 fold increase in ThioflavinS fluorescence might be due to Thioflaovin S binding caused by TDP-43 denaturation and aggregation.

### Validation of Functional TDP-43 Activity

**Figure 3:**
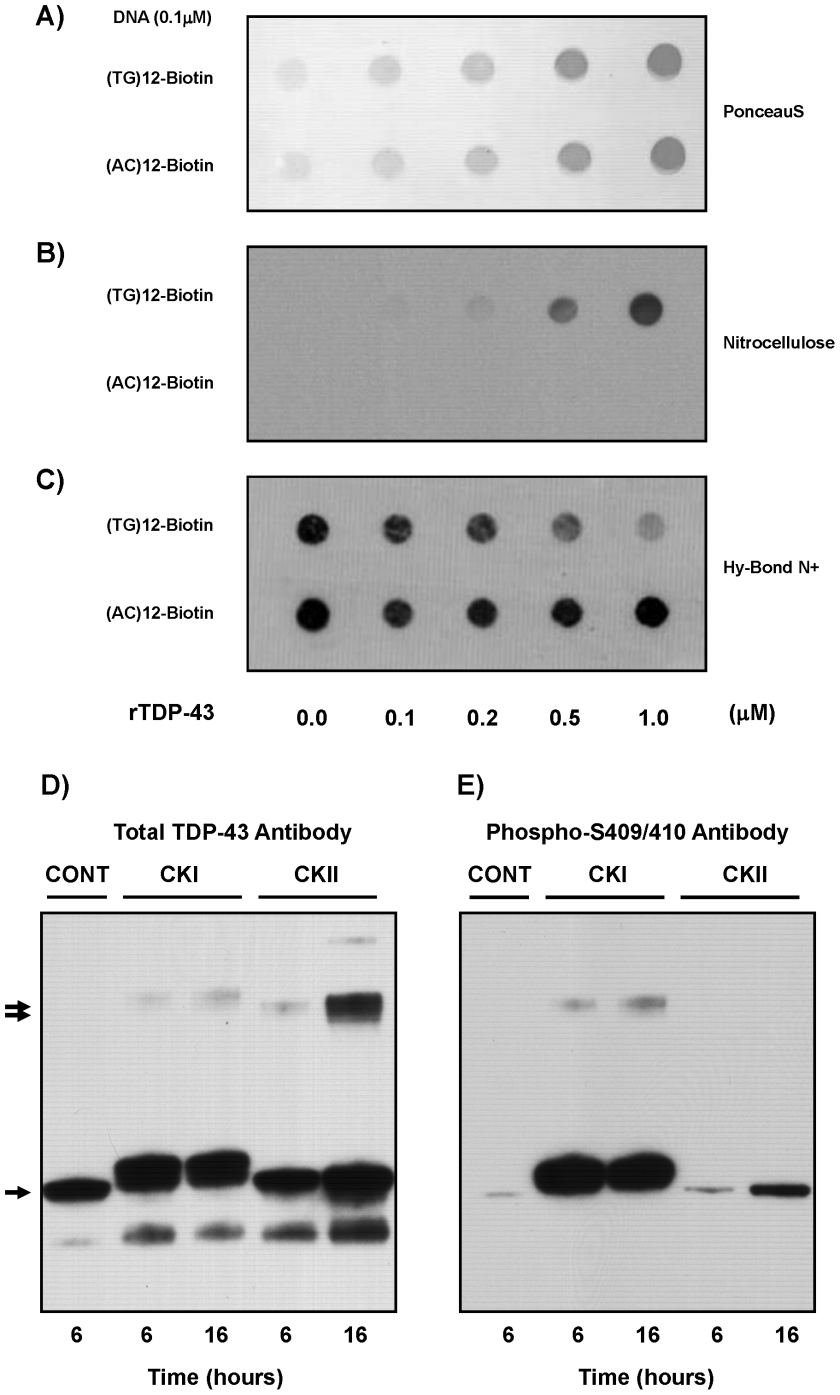
Functional Validation of Soluble rTDP-43. To verify that the rTDP-43 was functional, its ability to specifically bind repeated (TG)12 single stranded DNA compared to (AC)12 DNA was examined. A) Nitrocellulose was spotted with reactions of increasing concentrations of rTDP43 and fixed DNA probe to bind proteins as demonstrated by the Ponceau S staining. This nitrocellulose was placed on top of a DNA binding Hy-Bond N+ membrane which trapped the free biotinylated (TG)12 or (AC)12. B) The nitrocellulose retained the (TG)12 sequences indicating they were binding the rTDP-43 while C) biotinylated (AC)12 probes passed through to the Hy-Bond N+ for each amount of rTDP-43 spotted onto the nitrocellulose and the (TG)12 decreased in a dose dependent manner. Phosphorylation reactions demonstrate that the soluble rTDP-43 is a functional substrate for two known kinases. D) Phosphorylation with CKI leads to retardation of the monomeric rTDP-43 mobility in SDS-PAGE gels (single arrow) with little oligomer formation (double arrow) while CKII does not shift the apparent molecular weight of the monomer, but it leads to increased oligomer formation when total TDP-43 is monitored. E) Analysis of the phosphorylation by these kinases confirms both label the S409/410 sites, and CKI is more efficient than CKII.

Non-specific aggregation provides little detail as to the possible cellular activity of these preparations so to characterize the potential functionality of this soluble TDP-43 its ability to interact with known binding partners was examined. First, the ability to bind a physiologically relevant substrate was confirmed. TDP-43 is reported to specifically bind TG-repeated single-stranded DNA sequences, but not AC-repeated DNA sequences, and this assay has been used to demonstrate functionality using other tagged rTDP-43 preparations [24], [25]. As shown in **Figure 3A-C**, these soluble TDP-43 preparations bound the biotinylated (TG)12 probes when spotted onto nitrocellulose but not the (AC)12 probes which passed through the nitrocellulose to the DNA-binding nylon membrane. Furthermore, as TDP-43 is a phosphorylated protein *in vivo*, reactions were set up using CKI or CKII, for which TDP-43 is a known substrate. These results demonstrated this preparation of rTDP-43 is recognized by these kinases and is in a conformation that permits phosphorylation. As shown in **Figure 3D**, incubation with ATP and CKI led to retarded mobility of the monomeric TDP-43, while incubation with CKII caused TDP-43 dimer formation. This was not observed when TDP-43 was incubated with heat inactivated kinases (data not shown). Moreover, this phosphorylation by CKI led to generation of the phosphorylation-specific epitopes at the S409/410 sites as previously reported and shown in **Figure 3E**, and the decreased phosphorylation reported at these sites with CKII was also confirmed [16]. The more robust dimerization presented here upon CKII was not previously observed, and it suggest this soluble untagged TDP-43 is more assembly competent and might be more fibrillogenic. This may be due to conformational changes resulting from the absence of potential TDP-43 binding partners, as the earlier report utilized enriched but not purified TDP-43. Alternatively these purified soluble untagged TDP-43 preparations may simply be better substrates for phosphorylation [16].

### Electron Microscopy of CKII Induced TDP-43 Aggregates

**Figure 4:**
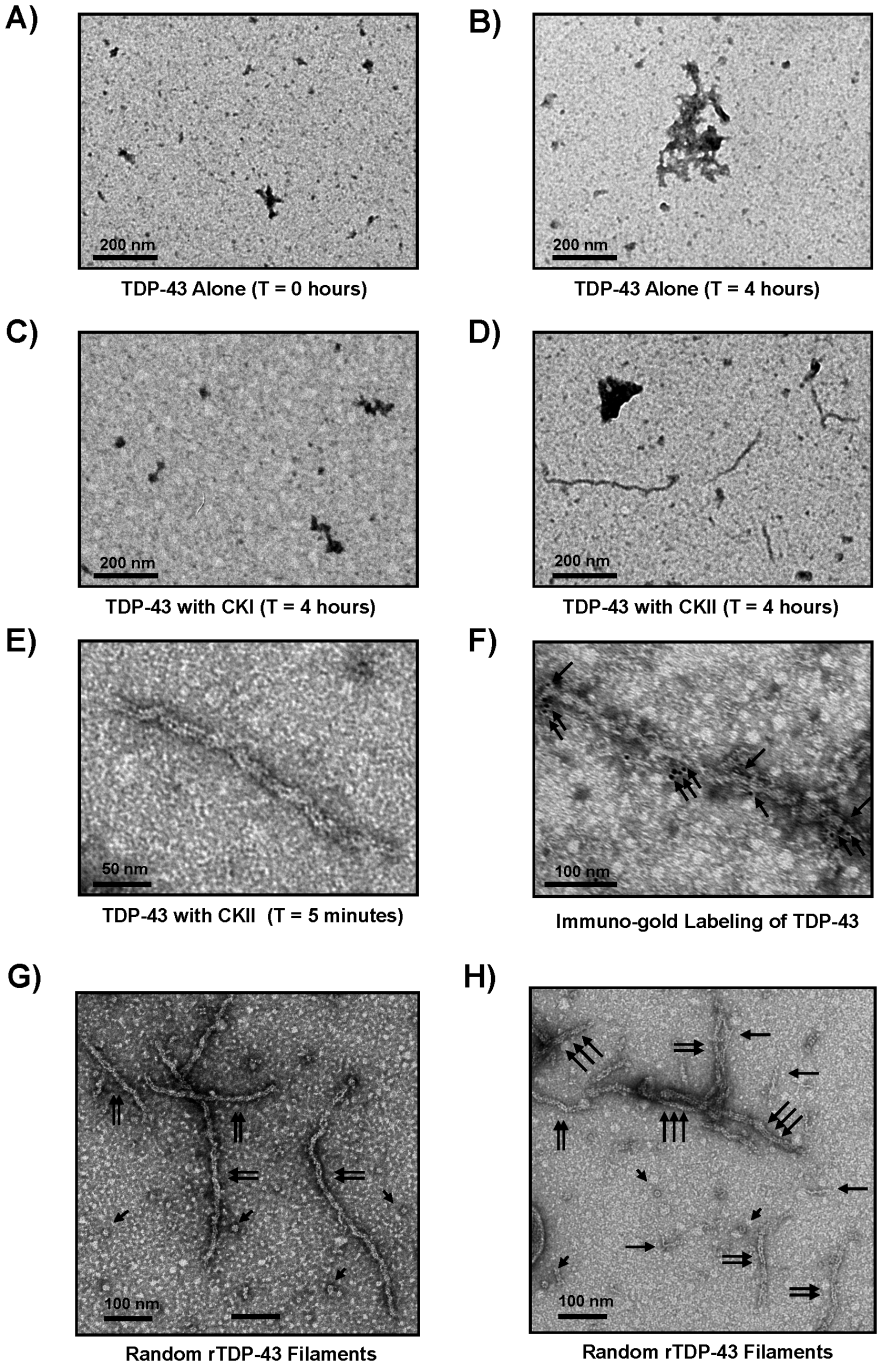
TDP-43 Filament Assembly after CKII Phosphorylation. A) Unphosphorylated rTDP-43 was prepared for electron microscopy immediately after high-speed clarification and no large aggregates or filamentous structures were observed. B) After 4 hours of incubation at 37°C without phosphorylation, no filamentous structures were not observed C) Similarly, soluble rTDP-43 samples phosphorylated with CKI for 4 hours did not form filaments, but D) the rTDP-43 incubated with CKII for 4 hours did polymerize. E) These filaments formed rapidly after CKII phosphorylation and could be detected at T = 5 minutes, and F) they could be immuno-gold labeled (arrows) with TDP-43 primary antibodies specific for the carboxy-terminus of TDP-43 (10 nm gold). G) The most common filaments observed after CKII phosphorylation of soluble rTDP-43 resemble randomly oriented FTLD 10–12 nm filaments (double arrows) shown above without a granular coating though numerous pore-like flowerette structures are also observed (arrowheads). H) Additionally smaller protofibrils (single arrows) and wider filaments (triple arrows) are occasionally found. The scales bar is in nanometers.

Previous reports demonstrated that phosphorylation of enriched rTDP-43 fractions with CKI would promote the polymerization of 10 nm wide filaments that could be immuno-gold labeled using anti-TDP-43 antibodies [16]. These filaments closely resembled those found in neuronal cytoplasmic inclusions and dystrophic neurites of FTLD and ALS cases with a reported width of 10–17 nm [22], but the effects the contaminants in these preparations had on assembly was not known. Electron microscopy of the soluble rTDP-43 purified here after high-speed clarification and before CKI or CKII phosphorylation indicated that there was little aggregation and no detectable filament assembly as shown in **Figure 4A**. Similarly no filaments were observed after CKI phosphorylation and incubation at 37°C for 4 hours though numerous filaments were observed after CKII incubation for this same time as shown in **Figure 4B–D**. Again, this was not observed when the CKII was heat inactivated before incubation with TDP-43 indicating this TDP-43 polymerization is phosphorylation dependent (data not shown). In fact, CKII phosphorylation of TDP-43 promoted filament formation as quickly as the kinase could be added to the reaction and the EM grids could be prepared, and these structures could be immuno-gold labeled with antibodies against the carboxyl terminus of TDP-43 as shown in **Figure 4E–F**. This is in contrast to the findings reported with enriched rTDP-43 polymerized after incubation with CKI, and again this may be due to effects of contaminating factors in those unpurified rTDP-43 samples [25]. Closer examination of the aggregation properties of the filamentous structures formed here with soluble rTDP-43 demonstrated that the filaments formed were not laterally associated into bundles as observed in FTLD neuronal intranuclear inclusions shown in **Figure 1B** but rather more closely resembled disassociated filaments without the granular coating reminiscent of FTLD-TDP accumulations as shown in **Figure 1D–E**. In addition, a variety of other, non-filamentous structures were also observed, including pore-like structures (arrowheads) typically found with other fibrillogenic amyloid proteins [41]. These structures were also observed in the presence of single protofibrils (single arrows) and 10–12 nm wide filaments (double arrows), which were the most prevalent, in addition to the wider filament structures (triple arrows). However, given that these wider filament structures were not observed until later time-points and were relatively scarce, this may indicate they are structurally composed of the typical 10–12 nm wide filaments as shown in **Figure 4G–H**.

### Characterization of TDP Filament Strcutures following CKII Phosphorylation

**Figure 5:**
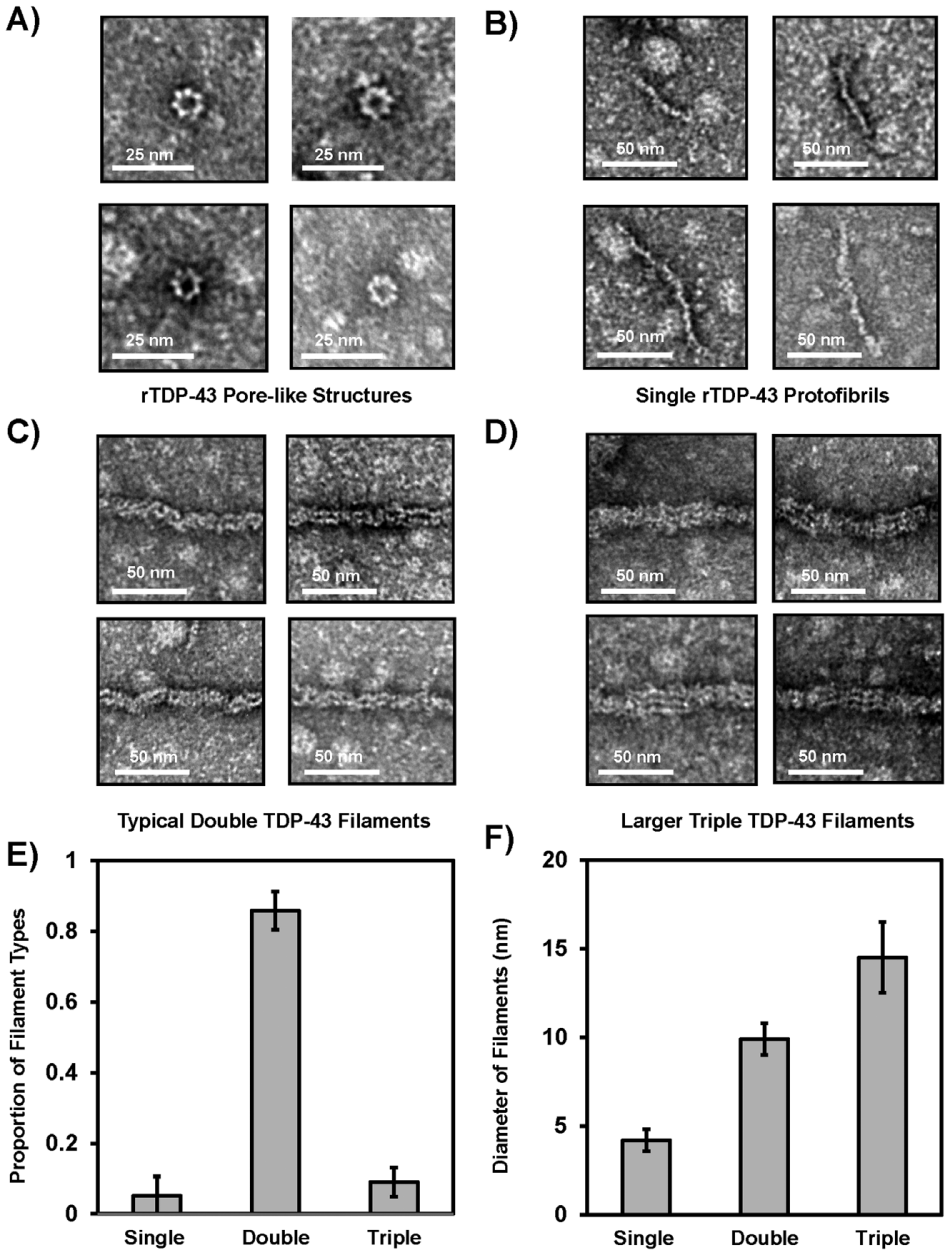
TDP-43 Polymerization into Structures Resembling FTLD Filaments. A) Numerous pore-like structures are detected before and after CKII phosphorylation. These putative pores have an average outer diameter of 13.5 +/− 1.2 nm and an inner opening of 6.2 +/− 0.8 nm. These were observed in all the reactions. B) Single filament protofibrils are less commonly observed, and they have an average diameter of 4.2 +/− 0.6 nm. E) The mostly commonly observed filaments are 9.9 +/− 0.9 nm and appear most similar to those detected in FTLD-TDP patients. These appear to be composed of two smaller tracks resembling the protofilaments, but the vast majority of the structures have clean ends indicating that are not formed by assembly of the smaller preformed protofibrils. F) Similarly, the larger 14.5 +/− 2.0 triple filament structures are rarely observed with splayed ends displaying smaller two track filaments or protofibrils. G) The double track 9.9 +/− 0.9 nm structures accounted for nearly 90% of the filaments detected in images captured for filaments characterization. H) The near integral multiples of the widths of these filaments indicate they may share a common structural protofilament even if the larger diameter filaments do not assemble from the smaller fibrils.

Aggregation of rTDP-43 preparations is well documented, but the presence of pore-like structures has not been reported. As shown in **Figure 5A**, soluble rTDP-43 formed pore-like units similar to those observed for other amyloidogenic proteins [41], and here the rTDP pores appear to consist of 7–8 subunits, with an outer diameter of 13.5 +/− 1.2 nm and an opening 6.2 +/− 0.8 nm across. These structures were also observed immediately after clarification of the soluble rTDP-43 and before incubation with the CKII (data not shown), indicating they formed spontaneously. These are smaller than the near 50 nm diameter oligomers found using tagged-TDP-43 [24]. The 4.2 +/− 0.8 nm wide protofibrils, most commonly observed 9.9 +/− 0.9 nm double track filaments, and the larger 14.5 +/− 2.0 nm filaments were only detected after CKII phosphorylation as shown in **Figure 5B–D**. The narrowest 4.2 nm filaments or protofibrils shown in **Figure 5B** resemble the tracks observed in the larger 9.9 nm and 14.5 nm filaments that were detected, but it is not known if these protofibrils actually assembled into the larger filaments, or if they have an incompatible subunit structure that precludes their assembly into those larger filaments. The most common 9.9 nm wide filament structures detected in all of the experiments performed in the current studies accounted for greater than 98% of the filament types observed with CKII phosphorylation (data not shown). As shown in **Figure 5C**, these 9.9 nm filaments occasionally appear as two tracks of the narrower protofilaments, or as a randomly twisted arrangement of these tracks. Similarly, the largest diameter 14.5 nm wide filament structures as shown in **Figure 5D** also appear to be comprised of protofilament tracks, but it is not known if these too form from the assembly of the smaller 4.2 nm protofibrils or the most commonly detected 9.9 nm filaments. In images gathered for the width measurements of the single and triple track filaments, 90% of the filaments were 9.9 nm double track structures (**Figure 5E–F**).

### Quantitation of the Assembly Properties of Soluble rTDP-43

**Figure 6:**
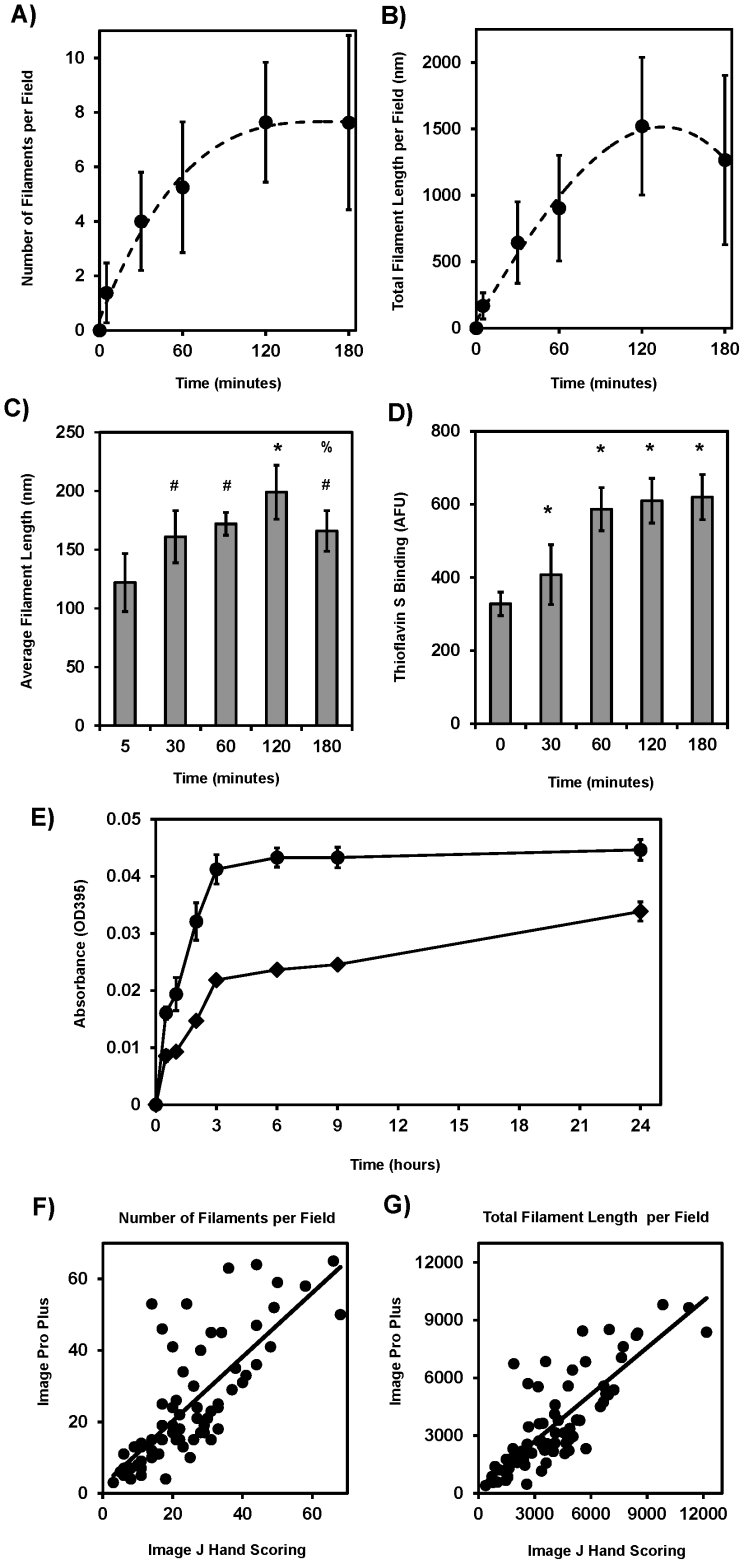
CKII Promotes TDP-43 Filament Assembly. A) Manual quantitation of the electron micrographs using NIH Image J to identify and hand measure the length of each filament indicates the number of filaments reaches steady state at T = 120 minutes. B) This is reflected in the total summed filament length per image which also reaches equilibrium at T = 120 minutes. C) The average length of these filaments does appear to grow from the thresh hold limit of 80 nm at T = 5 minutes to nearly 200 nm at T = 120 minutes. Here the # indicates p<0.10 and the * indicates p<0.01 compared to T = 5 minutes values. The % indicates p<0.10 for the T = 120 and T = 180 minute lengths perhaps indicating a length redistribution. D) Similarly, there is a modest increase in Thioflavin S fluorescence with the addition of CKII that is 1.9 X higher at T = 180 minutes than the monomeric rTDP-43 assayed at T = 0 minutes, and again the * indicates p<0.01 significance. This was a larger increase than the 1.3 X fold Thioflavin S fluorescence increase detected due to aggregation alone presented earlier. E) Similarly there was only a modest 1.3 x fold increase in turbidity between the buffer controls (diamonds) and the CKII treated TDP-43 samples (circles) again indicating quantitation of the electron micrographs provides the most accurate representation of the soluble rTDP-43 polymerization process. F) Image Pro plus was able to identify a very similar but lower numbers of filaments compared to Image J manual counting as indicated by a slope of less than 1.0 in the best fit line where IPP  = 0.898 (NIJ) + and R^2^ = 0.578. G) Also the total filament length was slightly underestimated using Image Pro Plus where IPP  = 0.812 (NIJ) + 265 and R^2^ = 0.664. This provides a significant increase in counting efficiency even with the manual IPP masking.

It is difficult to assess the true filament assembly properties of TDP-43 using traditional high-throughput techniques as the filaments are reported to be predominantly ThT- negative and TDP-43 aggregates into amorphous precipitates so readily it precludes the use of light scattering or pelleting assays that rely on ultracentrifugation [25], [42]. Fortunately, in the current study, the filamentous structures observed with this soluble rTDP-43 following CKII phosphorylation appear to be rarely associated with other TDP-43 filaments through lateral interactions that form bundles as when denatured rTDP-43 assembled into 3 nm wide filaments following simple agitation or as seen in FTLD NIIs in **Figure 1B** [25]. Accordingly, and as is routinely done with Tau, quantitation of electron micrographs was used to assess the assembly characteristics of soluble rTDP-43 after phosphorylation [43]. This methodology does not provide a measure as to the absolute amount or proportion of material that polymerizes into filaments, but this semi-quantitative evaluation via EM does allow for the comparison of seeding rates into filaments, the average lengths of the filaments formed, as well as the amount of total polymerized material per field. As shown in **Figure 6A–B**, rTDP-43 began forming filaments within 5 minutes of phosphorylation by CKII, and in fact it was not possible to collect a true T = 0 time point in the presence of active CKII, as filaments began to assemble as soon as the reagents were mixed and the samples prepared for EM. The CKII promoted seeding and filament assembly of rTDP-43 rapidly with half maximal seeding and total filament length per field reached by T = 60 minutes, and steady state levels observed by T = 120 minutes. There is some increase in the average filament length during these reactions beyond the minimal limit of 80 nm with maximum values reached by T = 120 minutes. The decrease in average filament length at T = 180 minutes was statistically significant (p<0.01) and may result from subunit exchange at the growing ends and aggregation or misfolding of the assembly competent subunits with increased time (**Figure 6C**). No significant changes in filament number, filament length or total filament length per field were detected when the incubations were extended to 360 or 960 minutes (data not shown). As shown in **Figure 6D**, thioflavin S fluorescence was increased significantly in parallel to the polymerization, but it was only 1.9-fold higher than CKII negative control. In comparison, gross aggregation was only increased 1.3-fold over CKII negative reactions as assessed by turbidity (**Figure 6E**). The comparison of manual assessment of filaments from electron micrographs using NIH Image J with computer-based assessment using Image Pro Plus software indicated that with the proper masking, some automation in counting can be achieved similar to that observed with recombinant Tau filaments, but each image does still have to be individually masked and inspected [43]. Additionally, the Image Pro Plus software did slightly underestimate the filament count (slope  = 0.90) and total length (slope  = 0.81) in comparison to NIH Image J (**Figure 6F–G**).

### Effects of Heat Shock Proteins on TDP-43 Polymerization

**Figure 7:**
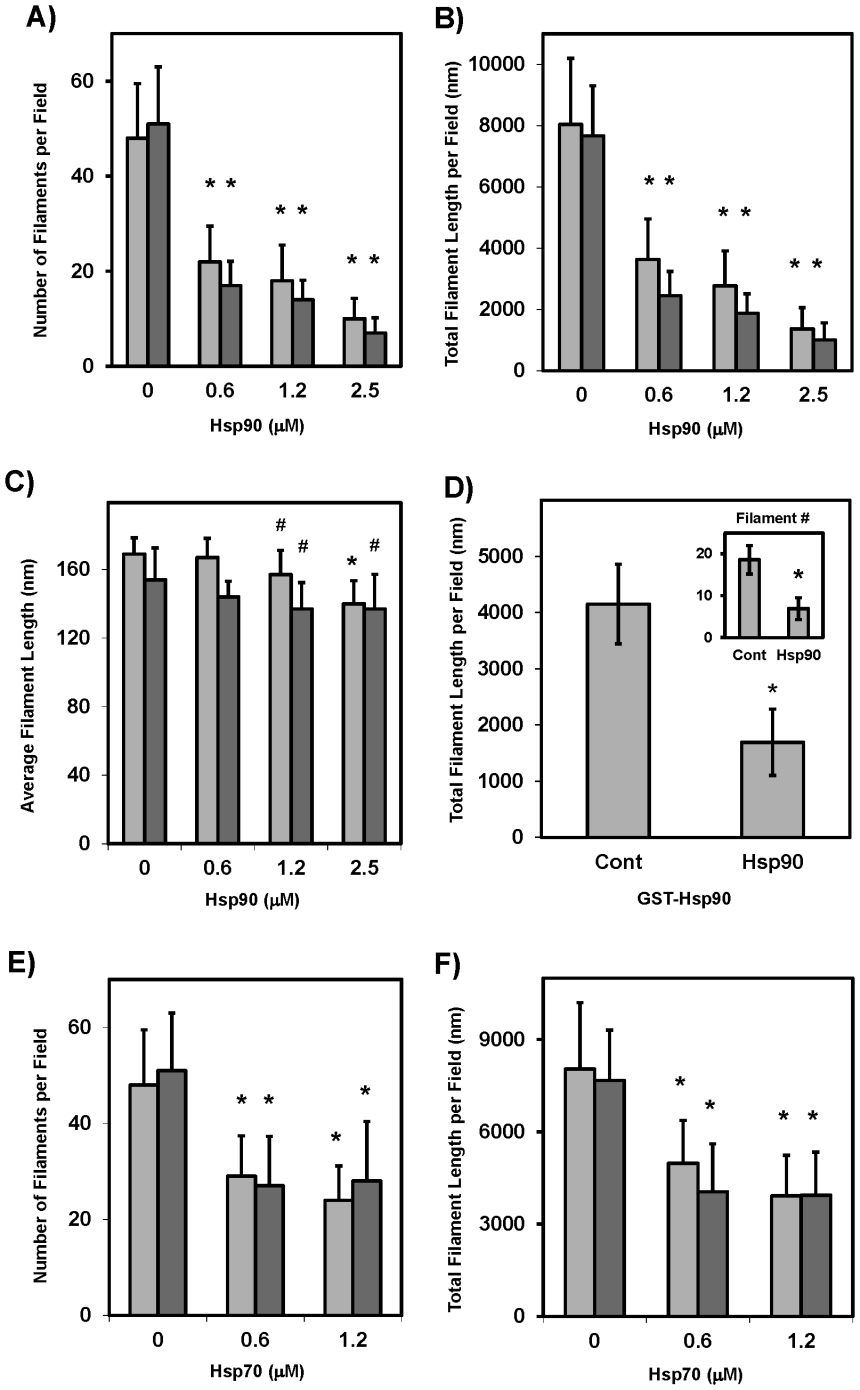
HSPs Inhibits CKII Mediated TDP-43 Filament Assembly. Hsp90 can inhibit the polymerization of tau, and TDP-43 is a known substrate for Hsp90. To examine the effects of Hsp90 on CKII mediated rTDP-43 filament assembly, 2.5 µM TDP-43 was polymerized for 60 minutes with CKII in the presence of 0.0–2.5 µM Hsp90. A) The number of TDP-43 filaments was dramatically reduced with increasing Hsp90, and this led to B) a substantial decrease in the total polymer mass even as C) the average length of the TDP-43 filaments was only slightly lowered. These data also confirmed Image Pro Plus (darker gray) provided reliable counting compared to the NIH Image J (lighter gray) allowing for larger fields to be counted. D) To confirm that these effects were not species dependent, GST-tagged recombinant human Hsp90 at 1.25 µM was incubated with 2.5 µM TDP as before and again the filament number and total filament length were reduced. Here the # indicates p<0.10 and the * indicates p<0.01 significance. Additionally, Hsp70 has been found to constitutively bind TDP-43 and is released after heat shock leading to TDP-43 aggregation. Hsp70 effects on CKII mediated rTDP-43 assembly support this observation as both the number of TDP-43 filaments (E) and the total filament mass (F) were decreased with increasing Hsp70.

**Figure 8:**
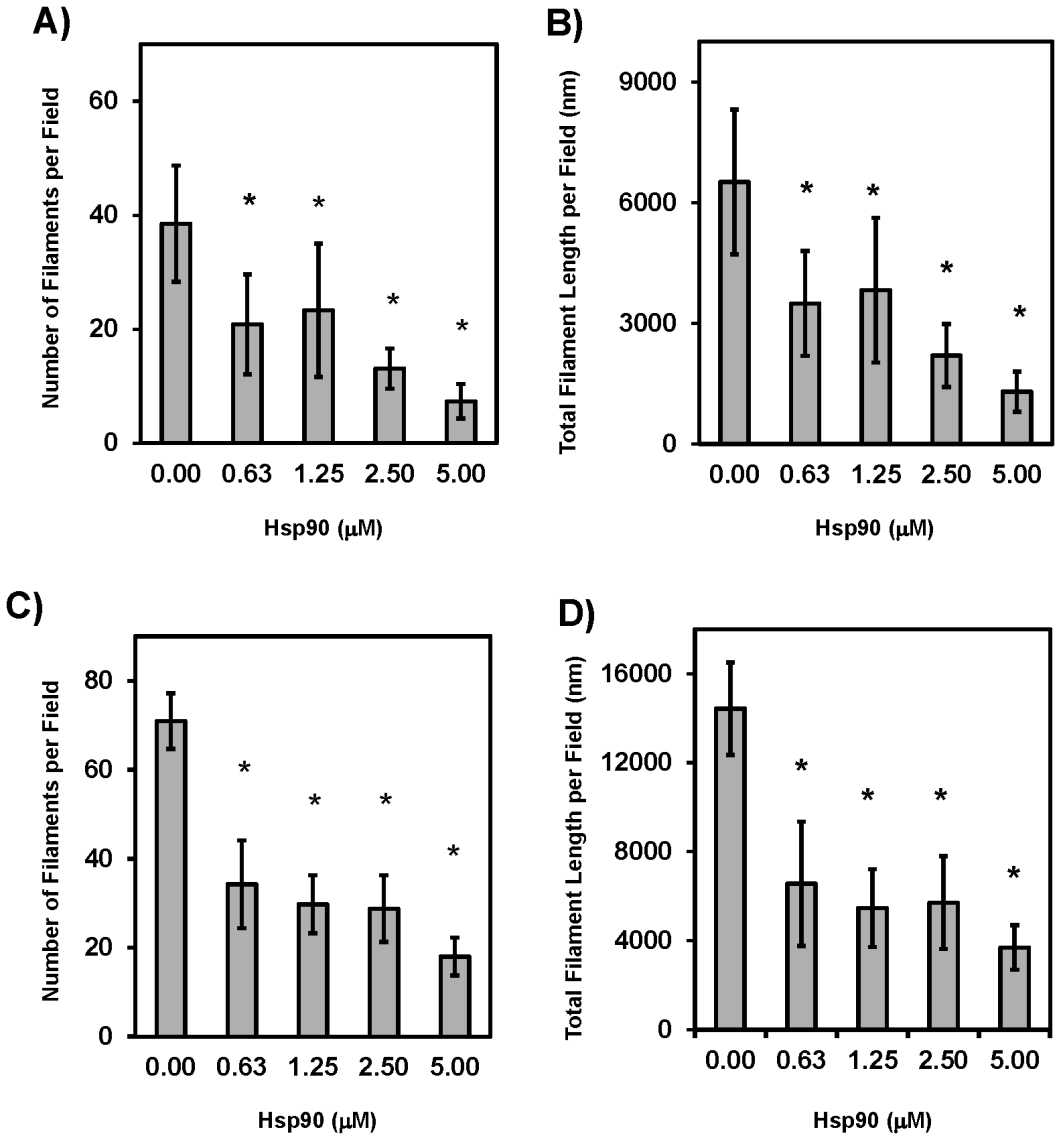
Hsp90 Promotes the Disassembly of Preformed rTDP-43 Filaments. To confirm Hsp90 was acting directly on the rTDP-43 and to determine if these Hsp90 binding sites on TDP-43 were masked upon polymerization, rTDP-43 was polymerized for 60 minutes with CKII in the absence of Hsp90. These preformed TDP-43 filaments were then incubated with increasing concentrations of Hsp90 and samples were assayed using Image Pro Plus from 0–16 hours. A) As rapidly as the Hsp90 could be added and the grids prepared for EM, the T = 5 minute measurements show the number of TDP-43 filaments was significantly reduced, and B) this translated into a robust dose dependent decrease in the total filament mass recorded per field. Furthermore these effects were only increased as the reactions then continued for 4 hours as demonstrated again by C) the filament number per field and D) the total filament mass per field. This remained throughout the time course. Here the * indicates p<0.01 significance.

Given that the same kinases are implicated in both the polymerization of TDP-43 and Tau filaments, and HSPs can inhibit the polymerization of Tau oligomers and filaments *in vitro* while also promoting Tau function *in vivo* [39], [44], [45], the effects of Hsp90 on CKII-promoted rTDP-43 polymerization into filaments were examined to determine if they were recognized as abnormal. As shown in **Figure 7A–B**, the addition of increasing amounts of Hsp90 was able to decrease the number of TDP-43 filaments that formed, thus leading to a concurrent decrease in the total amount of polymerized TDP-43 observed by EM. There was also a small but significant decrease in the average filament length as shown in **Figure 7C**, and this data set was used to validate the automation and efficiency gained using the Image Pro Plus (dark bars) compared to the NIH Image J hand scoring (light bars). Both techniques demonstrated that nanomolar levels of Hsp90 were able to inhibit the nucleation and polymerization of TDP-43, and the use of Image Pro Plus allowed for more area to be measured, resulting in a lower standard error and greater statistical significance. Additionally, recombinant human GST-tagged Hsp90 was shown to have a similar effect demonstrating that Hsp90-mediated inhibition of TDP-43 polymerization was not species-specific. These effects are believed to be direct as Hsp90 is known to activate CKII in cell culture [46], and this was not surprising as Hsp90 has been implicated in the chaperone mediated autophagy of TDP-43 that occurs when TDP-43 is mislocalized to the cytoplasm [36]. Additionally, Hsp70 has been shown to bind TDP-43 in the nucleus and can be is released by heat shock causing amorphous TDP-43 aggregation [11]. Experiments with inducible Hsp70 demonstrated similar effects on filamentous TDP-43 by retarding polymerization as shown in **Figure 7E–F**. Still the Hsp90 was significantly more effective than Hsp70 in inhibiting TDP-43 assembly as assayed by hand (p<0.05) or the Image Pro Plus (p<0.01). Furthermore, as HSP recognition motifs are thought to be in the core of the amyloidogenic Tau filament structures and may be inaccessible once polymerization occurs, the reversibility of TDP-43 filament formation upon addition of Hsp90 was assessed. Specifically, TDP-43 polymerization was initiated via phosphorylation in the absence of Hsp90 for 1 hour, and then these TDP-43 filaments were treated with increasing amounts of Hsp90 to assess the ability to recognize and promote the depolymerization of filamentous TDP-43. As shown in **Figure 8A–B**, Hsp90 was able to reduce the number and total polymer mass of the TDP-43 filaments in a dose-dependent manner within minutes of adding the Hsp90, and these effects were magnified when the incubation was then allowed to continue for 4 hours, indicating Hsp90 can interact with TDP-43 to slow or reverse phosphorylation-driven filament assembly (**Figure 8C–D**). These results provide further evidence that Hsp90 is acting on TDP-43 and not on CKII as the filaments were already formed before the addition of the Hsp90.

## Discussion

### Results on the Polymerization of Soluble rTDP-43 into FTLD-like Filaments

TDP-43 expressed using recombinant methods has been shown to be highly prone to aggregation, and this has limited research efforts directed at understanding how full-length wild-type TDP-43 can assemble into ordered filaments. Here, the data demonstrate the TDP-43 (**Figure 1**) can be expressed and purified in a soluble untagged form without the use of stabilizers, detergents or denaturants (**Figure 2**), and that this protein exhibits both the reported characteristics regarding aggregation and thioflavin binding when incubated alone and the specific interactions with repeated (TG)12 DNA ligands or CKI as a substrate (**Figure 3**). Furthermore, the data show that incubation of this soluble, full-length TDP-43 with CKII leads to its phosphorylation and subsequent polymerization into 9.9 +/− 0.9 nm wide filaments (**Figure 4**), and that these uncoated filaments closely resemble the most commonly observed 10–12 nm TDP-43 filaments in FTLD and AD patients that lack the granular coating and are randomly oriented (**Figure 1**). Examination of these TDP-43 structures using EM demonstrates that pore-like ringlets can form before CKII phosphorylation and that three prominent classes of TDP-43 filaments are detected following CKII phosphorylation (**Figure 5**). The larger filaments do not appear to be formed by association of the smaller diameter filaments or the pore-like structures. This is evidenced by the appearance of the most prevalent 9.9 nm double track filaments in parallel with the other filaments, and the observation that the 9.9 nm filaments usually have clean ends resulting from single subunit addition. Manual image analysis of electron micrographs using NIH Image J indicates TDP-43 filament assembly following CKII phosphorylation rapidly reaches equilibrium with regard to both filament seeding and total filament length within 2 hours, and this polymerization generates only slight increases in turbidity or thioflavin binding compared to unphosphorylated TDP-43 (**Figure 6**). Quantitative analysis of these TDP-43 filaments is amenable to automated counting using Image Pro Plus software as the filaments are uniformly distributed on the microscopy grids, and this allows for larger fields to be counted. Comparison of the data collected manually with NIH Image J and with automated Image Pro Plus demonstrates the results are correlated even if the masking needed for automation leads to recognition of slightly fewer filaments (**Figure 6**). In cell culture, Hsp90 is integral in the recognition of abnormal cytoplasmic TDP-43 aggregates, and it is a required component in the machinery that drives proteasomal TDP-43 degradation. Hsp90 may directly interact with consensus binding motifs on TDP-43 that are normally masked, and this might directly inhibit the polymerization of TDP-43 into filaments as reported for Tau indicating these filaments are abnormal. Indeed, the data show that Hsp70 and Hsp90 are potent inhibitors of the CKII-mediated polymerization of TDP-43 (**Figure 7**); and since Hsp90 is a known activator of CKII, this effect is most likely the result of Hsp90 directly interacting with TDP-43. Such findings would not necessarily imply Hsp90 can recognize polymerized TDP-43, but this appears to be true as Hsp90 can also promote the disassembly of preformed TDP-43 filaments. The data provide further evidence that Hsp90 directly interacts with TDP-43, and that its effects on TDP-43 polymerization are not mediated through Hsp90 interactions with CKII (**Figure 8**). These findings support our hypothesis that soluble untagged full length rTDP-43 can be expressed and purified to homogeneity, and that kinases like CKII involved in Tau polymerization can promote rTDP-43 assembly into filaments that are recognized as abnormal by the HSPs.

### Importance of Soluble rTDP Purification and Polymerization Methods

The development of biochemical techniques to produce soluble wild-type recombinant TDP-43 without protein tags or the use of chemical stabilizers and denaturants provides the cleanest system to study the effects of post-translational protein modification on TDP-43 function. This should permit the effects of modifications such as phosphorylation, acetylation and proteolysis on TDP-43 function, protein folding, stability, oligomerization and polymerization to be studied effectively without the concerns that arise from the use of artificial constructs or the chemical modifiers used in other studies. Furthermore the assembly of this soluble rTDP-43 into 9.9 +/− 0.9 nm diameter filaments that characterize the predominant 10–12 nm forms of filamentous TDP-43 inclusions observed in ALS, FTLD and AD provides the best resource to date for studying the effects of TDP-43 filament assembly on TDP-43 function and the ability of these filaments to sequester wild-type TDP-43 or other cellular components. These filaments should more accurately represent the structures found in disease because they are formed from purified TDP-43 which does not contain potential bacterial proteins that may have contaminated enriched TDP-43 fractions used in the only other study reporting 10 nm wide filaments with TDP-43 phosphorylation [16]. Furthermore, the TDP-43 filaments presented here are made from protein expressed and purified in the absence of a GST-fusion tag and glycerol. Subsequent differences in folding due to the tag may explain why the CKII promoted TDP-43 filaments here are more abundant by proportion than the mostly amorphous aggregates observed in preparations made from TDP-43 purified with a fusion tag, and any resulting TDP-43 mis-folding in those preparations may explain why the diameter of those filaments was 18.0 nm wide and were observed with a granular coating[24]. This is in contrast to the TDP-43 filaments prepared from HIS-tagged TDP-43 and purified using 6 M guanidine hydrochloride. Those preparations were also able to bind (TG)12 DNA probes specifically, but the filaments that formed upon agitation were tightly bundled and only 2.6 nm in diameter [25]. All of these recombinant studies differ from the recent publication of the TDP-43 filaments assembled from synthetic TDP-43 peptide. These studies were notable in that 46-mers of TDP-43 containing a putative prion-like sequence readily assembled into 7.7 nm high filaments that were thioflavin T positive and neurotoxic, but it is unlikely these form in disease and the 250 µM concentration used to promote polymerization is 100-fold higher than that observed here with CKII driven polymerization of soluble full-length TDP-43 [10]. Together, these studies suggest that the CKII-promoted TDP-43 filaments formed here represent the best tool for studying the ability of TDP-43 filaments to sequester wild-type TDP-43 or for making disease specific antibodies.

### Interpretation of CKII mediated TDP-43 Assembly Findings

The results reported here using CKII most closely recapitulate what might be expected when soluble and mislocalized, but not yet fibrillogenic, TDP-43 finds its way to the cytoplasm where it might be further modified and converted to polymerization competent forms. CKII levels are found to be up regulated in AD cases [47], [48], and this may explain why many AD cases also contain filamentous TDP-43 cytoplasmic inclusions. Evidence how this might occur can be garnered from studies on lentiviral expression of β-amyloid 1–42 in motor neurons of wild-type mice demonstrating that β-amyloid expression promotes the accumulation and oligomerization of endogenous TDP-43 with a concurrent increase in the expression of both CKI and CKII [49]. Furthermore this can be attenuated by parkin expression even in the presence of Tau pathology in wild-type or 3xTgAD mice suggesting the CKI and CKII activation and TDP-43 accumulation depend on of β-amyloid and not Tau [49]. This indicates CKII mediated polymerization of TDP-43 into filaments may represent a viable therapeutic target in AD if not ALS and FTLD also. Since numerous kinases have been implicated in the conversion of soluble Tau into hyperphosporylated AD p-Tau species that are fibrillogenic, this suggest any of these might also promote TDP-43 polymerization alone or as required for Tau in combination [38]. This is in apparent contradiction to several reports that TDP-43 phosphorylation may be defense mechanism to reduce it aggregation, but it is noted that these studies used truncated forms of TDP-43 and not full length protein [50], [51]. Aside from the fact that full length and truncated TDP-43 may behave differently, the importance of this distinction is still two-fold as increased solubility of full length TDP-43 upon phosphorylation might provide a window for TDP-43 polymerization into filaments before amorphous aggregation occurs spontaneously and truncation of full length TDP-43 might occur after aggregation. The latter has been indicated by the propagation studies showing TDP-43 cleavage induced through seeding mirrors the truncation observed in the TDLD disease strain used, and presumably these signature degradation patterns result from the morphologically distinct TDP-43 filaments that are formed and their differing susceptibilities to subsequent proteolysis [27]. In light of the caveats in those studies, the findings presented here that demonstrate CKII does promote the assembly of rTDP-43 into 9.9 nm wide filaments characterizing many TDP-43 proteinopathies suggests CKII phosphorylation of TDP-43 may be pathologically relevant and have therapeutic potential.

### Implications of Hsp90 Mediated TDP-43 Filament Depolymerization

The recognition and disassembly of these CKII induced rTDP-43 filaments by Hsp90 suggest the phosphorylated TDP-43 is abnormal and contains exposed Hsp90 binding sites further strengthening the argument that these filaments are pathologically relevant. The generation of filamentous TDP-43 should facilitate the determination of the role particular TDP-43 filaments play in sequestering normal TDP-43 intraneuronally or through cell-to-cell propagation interneuronally, and the functional consequences and cellular responses of this conversion of normal TDP-43 into filamentous aggregates can be examined. Polymerized rTDP-43 filaments could be introduced into cells using protein transfection reagents, and the ability of filamentous TDP-43 to recruit normal endogenous TDP-43 from the nucleus could be determined using immunofluorescence, while the functional effects of seeding could be assessed through the examination of TDP-43 RNA client transcripts [25]. Furthermore, the Hsp70 and Hsp90 response to these actual filamentous TDP-43 inclusion can be determined, as well as the ability of Hsp90 inhibitors to promote TDP-43 filament disassembly and degradation. It has been demonstrated that amorphous TDP-43 aggregates promotes induction of Hsp70 expression in cell culture and that Hsp90 inhibitors can promote the degradation of normal endogenous TDP-43 [31], but how these HSPs affect the more pathologically prevalent filamentous TDP-43 is still not known. The results here coupled with the report that Hsp40and Hsp70 may normally bind some TDP-43 and prevent its aggregation in the nucleus suggests increasing HSF1 expression might be beneficial [11]. Since Hsp90 is also known to bind TDP-43 [36] and these experiments demonstrate Hsp90 can inhibit and reverse CKII mediated TDP-43 assembly into filaments, it suggests both traditional Hsp90 inhibitors that promote proteasomal mediated Hsp90 client degradation with increased HSF1 expression and those that inhibit the Cdc37/Hsp90 interaction and promote autophagic clearance might be provide another therapeutic strategy preventing filamentous TDP-43 lesions [36]. Intriguingly, CKII phosphorylation of Cdc37 is thought to be essential for Cdc37/Hsp90 chaperoning function indicating CKII activation may stabilize the Cdc37/Hsp90 clients as observed in multiple myeloma, and hence these clients may additionally be targeted for degradation by the use of CKII inhibitors that block Cdc37 phosphorylation [52]. The ability to generate TDP-43 filaments and transfer them into cells should allow for these potential Hsp90 mediated therapeutic avenues to elucidated with regard to the clearance of filamentous TDP-43 lesions as well as furthering our understanding of the roles TDP-43 filament sequestration and propagation play in pathogenesis whether though loss of TDP-43 function or the isolation of TDP-43 mRNA clients and other essential cellular RNAs and related binding proteins. This would represent a significant step forward in determining the effects of TDP-43 filamentous inclusions in the cell and whether they are intrinsically harmful.

### The Significance of the Findings Regarding TDP-43 Aggregation in Disease

Filamentous TDP-43 inclusions form pathological lesions in some of the most prominent and debilitating neurodegenerative diseases including the motor neuron disease ALS where TDP-43 mutations are causative [2], as well as the most prevalent dementias FTLD and AD [19], [20]. These abnormal TDP-43 accumulations can be found as both amorphous aggregates and more frequently highly-ordered filamentous structures present within the nucleus, cytoplasm or dystrophic neurites, and collectively all inclusion variants are implicated in the loss of normal TDP-43 function and potential toxic gain of functions [22]. This presumably can occur through the direct conversion of normal TDP-43 into these aggregation-prone forms, and any concurrent loss of functional TDP-43 may be accelerated by the sequestration of normal TDP-43 into the abnormal lesions [27]. These lesions may affect either normal TDP-43 RNA processing function or act to isolate other essential mRNA transcripts and proteins in the cell. Ultimately, the loss of TDP-43 function as well as toxic gains of function might occur anywhere along the process where modifications affect translocation, aggregation, sequestration or propagation, and this punctuates the critical importance in understanding the factors that control TDP-43 aggregation and polymerization. Efforts to determine the mechanisms of TDP-43 filament assembly have been limited by the absence of reported methodology for producing soluble, recombinant wild-type TDP-43 that is not tagged, as well as the development of paradigms for driving TDP-43 polymerization into 10 nm wide filaments that are amenable to quantitation and most frequently observed in disease. This report establishes these methods and demonstrates the polymerization of 10 nm TDP-43 filaments upon CKII phosphorylation is recognized as abnormal and can be reversed by Hsp90 further supporting their pathological relevance. The development of these methods to create synthetic TDP-43 filaments should accelerate research not only investigating the relationship between TDP-43 aggregation and the loss of its normal function or any toxic gain of function but also as tools for generating antibodies to filamentous TDP-43 for clinical diagnosis, pathological confirmation of disease and possibly treatment.

### Conclusions from these Research Findings

The polymerization of soluble wild-type TDP-43 into 10 nm filaments characteristic of ALS, FTLD and AD upon CKII phosphorylation suggests this signaling pathway is involved in disease pathogenesis. The novel techniques described here for untagged TDP-43 purification and the unprecedented demonstration that CKII can drive TDP-43 filament assembly that outpaces its generic aggregation indicates that free TDP-43 deposition may be controlled in a disease specific manner resulting from the confluence of multiple independent mechanisms. The identification of CKII as a kinase that can promote TDP-43 assembly without any further modification means this pathway may be susceptible to therapeutic modification in the treatments of these diseases, and the binding of Hsp90 to TDP-43 following phosphorylation by CKII demonstrates this protein is recognized as abnormal. The potential of targeting this CKII phosphorylated TDP-43 though pharmacological manipulation of the Hsp90 chaperones system is enhanced dramatically by the demonstration Hsp70 and Hsp90 can both inhibit CKII mediated TDP-43 filament assembly and promote the depolymerization of TDP-43 filaments once formed. Indeed CKII phosphorylation of aggregates composed of only carboxy-terminal fragments of TDP-43 has been shown to target these for disassembly in cell culture, but it was not determined whether the HSPs then promote any beneficial TDP-43 degradation also [50]. Sarkosyl insoluble fractions from FTLD-TDP cases contain both full length and degraded phosphorylated TDP, and this may complicate assessing the exact role of CKII in pathogenic TDP-43 deposition [53]. In fact, CKII phosphorylation might promote the assembly of full length TDP-43 and then later be responsible for its degradation when it has been acted on by proteases to generate the phosphorylated fragments of TDP-43. Collectively these data imply both CKII phosphorylated forms of TDP-43 may be susceptible to proteasomal degradation upon Hsp90 inhibition even after depositing as filamentous lesions in these diseases, and this would increase the window for therapeutic modulation of CKII activity beyond the preventative stages. Furthermore it suggests further exploration of CKII phosphorylated TDP as a disease marker in ALS and FTLD is warranted.

## Materials and Methods

### Ethics Statement

Human postmortem brain tissue was provided by the Mayo Clinic Brain Bank (http://www.mayo.edu/research/departments-divisions/department-neuroscience-florida/brain-bank). Written informed consent from the donor or the next of kin was obtained for brain autopsy and the use of tissues for research purposes. All studies involving autopsy materials were approved by the Mayo Foundation Institutional Review Board. All animal procedures were approved by the Mayo Institutional Animal Care and Use Committee (Protocol # A30911) and are in accordance with the National Institutes of Health Guide for the Care and Use of Laboratory Animals (NIH Publication 80–23).

### Reagents, Chemicals and Antibodies

Protease Inhibitor Cocktail (PIC, # 539131) was purchased from Fisher Scientific Incorporated in Pittsburg PA, and Phosphatase Inhibitor Cocktail II (#5726) and III (#P0004) were from Sigma Corporation in Cream Ridge NJ. DNAase (#5025), ADP-agarose (#A2810) and dithiothreitol (#D0632) were from Sigma-Aldrich Corporation in St. Louis MO. Bicinchoninic acid reagents (BCA, #P23223/P23224) and bovine serum albumin (BSA, #P23209) were from Pierce Thermo Fisher Incorporated in Rockford IL. Electron microscopy carbon/formvar coated 400 mesh copper grids (#FCF400-Cu) were purchased from Electron Microscopy Sciences in Hatfield PA. The kinases used were obtained as follows: Casein Kinase I (#P6030S) and Casein Kinase II (#P6010L) were from New England Biolabs in Ipswich MA. Streptavidin labeled horse radish peroxidase (#016-030-084) was from Jackson ImmunoResearch Laboratories in West Grove PA.

### Expression and Purification of Recombinant TDP-43

Human TDP-43 cDNA was cloned into pET30b for expression under the T7 promoter. Overnight 3 ml culture were prepared in Luria broth with 20 µg/ml kanamycin using frozen glycerol stocks and grown overnight with shaking at 37°C. These were used to inoculate 300 ml of LB media again with 20 µg/ml kanamycin, and these were grown with shaking at 37°C until the OD600 reached 0.5 absorbance. These cultures were then chilled at 4°C for 30 minutes without shaking before induction and then transferred to a 16°C shaker for overnight induction with 0.3 µM Isopropyl β-D-1-thiogalactopyranoside. Cells were then collected by centrifugation, washed 1x in Tris-buffered saline (10 mM Tris buffer, 140 mM NaCl, pH 7.4), collected and frozen at −80°C. These cells were then resuspended in lysis buffer (10 mM Hepes, 100 mM NaCl, 1 mM MgCl_2_, 1 mM dithiotreitol, 1 mM PMSF, 1x PIC and 21 units/ml DNAase, pH 7.4) and lysed by probe sonication for 30–90 seconds on ice at 50% power in a Bronson sonicator. Cell debris and inclusion bodies were removed by centrifugation at 12,000×g for 20 minutes, and saturated ammonium sulfate solution was added to 15% saturation while on ice for 10 minutes. The precipitated proteins were collected by centrifugation at 12,000×g for 10 minutes, and the pellets were resuspended in 8 ml of assembly buffer (AB, 10 mM Hepes, 100 mM NaCl, 1 mM PMSF, pH 7.4) by rocking at 4°C for 30 minutes. The samples were clarified at 12,000×g for 10 minutes and dialyzed 3 times against 200 volumes of assembly buffer. The rTDP-43 was then clarified again at 12,000×g for 10 minutes, and then aliquotted and snap frozen in liquid nitrogen before storage at −80°C. Before use, the soluble rTDP-43 was collected after clarification at 100,000×g for 37 minutes, and the protein concentration was determined by BCA analysis with BSA standards. Purity was analyzed by SDS-PAGE and Coomassie brilliant blue staining.

### Turbity Assays

TDP-43 was incubated at 37°C at 2.5 µM with 3 seconds of shaking between reading as the turbidity was assessed via OD395 reading in 96 well plates using a SpectraMax plate reader for 120 minutes with measurements each minute [54].

### Purification of Heat Shock Protein 90

Hsp70 and Hsp90 proteins were purified from mouse brain tissue as described elsewhere making use of ADP-agarose and anionic exchange chromatography [55]. Some experiments were repeated using the same purification protocol on recombinant human to clean GST-tagged Hsp90 following a glutathione-sepharose chromatography step. The mouse brain homogenates used for these preparations were generated from tissues that had been collected immediately after cervical dislocation and flash frozen in liquid nitrogen to minimize changes in post-translational modification of proteins that might result from other methods of sacrifice. These were stored at −80°C until used.

### DNA Binding Protocol

Binding reactions with 0.1 µM biotinylated (TG) 12 or (AC)12 DNA probes were incubated with soluble 0.1–1.0 µM TDP-43 proteins in TDP assembly buffer for an hour at 37°C. The incubation reaction was then filtered through a pretreated nitrocellulose membrane overlaid on an assembly buffer equilibrated nylon membrane in a 96-well slot blot apparatus. After washing with 3 ml of assembly buffer per well, the bound biotin-labeled (TG)12 or (AC)12 DNA probes were cross-linked to the membranes by shortwave ultraviolet radiation for 2 minutes on each side. The membranes were then blocked with 5% milk in TBS with 0.1% Triton X-100 for 60 minutes and then incubated with streptavidin-HRP diluted at 1∶5000 for 60 minutes at room temperature. The biotin-labeled DNAs on the membranes were developed with standard ECL reagents. To reduce nonspecific single-stranded DNA binding to the nitrocellulose membranes that was not TDP-43 dependent, the membranes were presoaked for 60 min in 0.4 M KOH, rinsed in water and then equilibrated in assembly buffer for 30 minutes before slot blotting. The nylon membranes were not treated, but they were also incubated in assembly buffer for 30 minutes prior to the filtration [25].

### Phosphorylation and Assembly Reactions

Recombinant TDP-43 filament phosphorylation assembly reactions were set up in 1 x AB buffer supplemented with 1x kinase buffer (KB, 20 mTris, 50 mM KCl, 10 mM MgCl_2_, pH 7.5) as recommended by the kinase provider. These reactions contained 2.5 µM rTDP-43 supplemented with 500 µM ATP and the kinase indicated. Control reactions used heat denatured kinases or 1 x KB in substitution of the kinase volume. Typically 1 µl of kinase was used in a 30 µl reaction. All reactions were incubated at 37°C without shaking for the time indicated, and then these were immediately prepared for western blotting or electron microscopy as described. Experiments to examine the effects of Hsp90 on rTDP-43 assembly were carried out as described except Hsp90 was added at 0.0–2.5 µM for the duration of the phosphorylation assembly reactions. Experiments to examine the effects of Hsp90 on pre-assembled rTDP43 filaments were carried out as described except the concentration of rTDP-43 and kinases and ATP were proportionally scaled up by a third to 3.25 µM rTDP-43, 1.3 µl CKII and 667 µM ATP and incubated as described for 2 hours to reach maximal filament assembly before adding 4× Hsp90 so the final rTDP-43 concentration was again 2.5 µM and the Hsp90 was 0.0–5.0 µM. These disassembly reactions were then incubated as described for 0–4 hours, and grids for EM were taken during this time as indicated.

### Western Blotting

Samples were run on 10% SDS-PAGE Tris-glycine gels and transferred to nitrocellulose membranes at 200 mA constant current for 2.5 hours. Blots were subsequently blocked in 5% non-fat dry milk suspended in 1 X TBS + 0.1% Ttriton X-100 (TBST) for 60 minutes. Blots were then incubated overnight with either total TDP-43 antibody or phosphorylation specific S409/410 TDP-43 antibodies. Blots were developed using ECL Plus reagents and traditional film.

### Tissue Fixation

Tissue was collected from the parahippocampal gyrus of formalin-fixed brains from pathologically confirmed cases of FTLD-TDP. All tissue was dehydrated in ethanol and embedded in LR White [56]. Thin sections collected on Formvar-coated-nickel grids were floated with the section-side down on 2 ml of citrate buffer, pH 6, in a 100°C oven for 10 min, and cooled to room temperature prior to immunogold EM. The primary antibodies used were as follows: polyclonal antibody to TDP-43 (1∶20; ProteinTech Inc., Chicago, IL) and monoclonal antibody to TDP-43 (1∶30, Abnova, Taipei, Taiwan). To detect the location of the primary antibodies, 10 nm gold-conjugated secondary antibodies were used (1∶20; Amersham/GE Healthcare, Piscataway, NJ).

### Electron Microscopy

Reaction samples were diluted 4x in 1XTBS, and 10 µl was adsorbed onto carbon/formvar-coated 400 mesh copper grids (EM Sciences) for 45 seconds. These were then stained with 2% uranyl acetate for 45 seconds, and the grids were examined with a Philips 208S electron microscope (Philips, Hillsboro, OR). For quantification, twelve images were collected randomly at three predetermined coordinate locations that remained fixed throughout these studies.

### Filament Analysis

These 12 images were collected at 5,000× magnification, and the average filament number, average filament length and total filament length per field was measured manually using Image J freeware [57]. The length bar was also measured so these lengths could be converted into nanometers. Alternatively, the images were analyzed using Image Pro Plus Version 7.0 software. The background was threshold-adjusted so only the aggregates were marked and then the filaments were selected for counting by setting limits on the minor axis (2–20) and the length (15–100,000). Images had to be adjusted manually to account for variations in the staining and to ensure only proper filaments were scored. This was clearly more efficient than manually tracing each filament and using the Image J software to score the filament number and lengths.

### Thioflavin S Binding

TDP-43 filament assembly was monitored by fluorescence of thioflavin S binding using a Cary Varian Eclipse Spectrofluorimeter (Walnut Creek, CA) with an excitation wavelength of 440 nm and a slit width 10 nm, and emission spectrum collected from 460–600 nm and a 10 nm slit width. Measurements were performed at room temperature after incubating 30 µl of TDP-43 phosphorylation reaction with 90 µl of 0.006 mg/ml thioflavin S for 30 minutes. Negative controls included buffer alone, kinase and buffer, and reactions containing TDP-43 alone, but each with thioflavin S as above. Thioflavin S binding intensity was measured by integrating the curve between the ranges of 460–600 nm using the Cary Eclipse Scan software.

## References

[1] Ayala YM, Pantano S, D'Ambrogio A, Buratti E, Brindisi A, et al (2005) Human, Drosophila, and C.elegans TDP43: nucleic acid binding properties and splicing regulatory function. J Mol Biol 348: 575–588. 15826655 10.1016/j.jmb.2005.02.038

[2] Neumann M, Sampathu DM, Kwong LK, Truax AC, Micsenyi MC, et al (2006) Ubiquitinated TDP-43 in frontotemporal lobar degeneration and amyotrophic lateral sclerosis. Science 314: 130–133. 17023659 10.1126/science.1134108

[3] Sreedharan J, Blair IP, Tripathi VB, Hu X, Vance C, et al. (2008) TDP-43 mutations in familial and sporadic amyotrophic lateral sclerosis. Science 319: 1668–1672. Epub 2008 Feb 1628. 10.1126/science.1154584 PMC7116650 18309045

[4] Gitcho MA, Baloh RH, Chakraverty S, Mayo K, Norton JB, et al (2008) TDP-43 A315T mutation in familial motor neuron disease. Ann Neurol 63: 535–538. 18288693 10.1002/ana.21344 PMC2747362

[5] Rutherford NJ, Zhang YJ, Baker M, Gass JM, Finch NA, et al (2008) Novel mutations in TARDBP (TDP-43) in patients with familial amyotrophic lateral sclerosis. PLoS Genet 4: e1000193. 18802454 10.1371/journal.pgen.1000193 PMC2527686

[6] Gendron TF, Rademakers R, Petrucelli L (2012) TARDBP Mutation Analysis in TDP-43 Proteinopathies and Deciphering the Toxicity of Mutant TDP-43. J Alzheimers Dis 29: 29. 10.3233/JAD-2012-129036 PMC3532959 22751173

[7] Buratti E, Brindisi A, Giombi M, Tisminetzky S, Ayala YM, et al. (2005) TDP-43 binds heterogeneous nuclear ribonucleoprotein A/B through its C-terminal tail: an important region for the inhibition of cystic fibrosis transmembrane conductance regulator exon 9 splicing. J Biol Chem 280: 37572–37584. Epub 32005 Sep 37512. 10.1074/jbc.M505557200 16157593

[8] Fuentealba RA, Udan M, Bell S, Wegorzewska I, Shao J, et al. (2010) Interaction with polyglutamine aggregates reveals a Q/N-rich domain in TDP-43. J Biol Chem 285: 26304–26314. Epub 22010 Jun 26316. 10.1074/jbc.M110.125039 PMC2924052 20554523

[9] Liu-Yesucevitz L, Bilgutay A, Zhang YJ, Vanderweyde T, Citro A, et al (2010) Tar DNA binding protein-43 (TDP-43) associates with stress granules: analysis of cultured cells and pathological brain tissue. PLoS One 5: e13250. 20948999 10.1371/journal.pone.0013250 PMC2952586

[10] Guo W, Chen Y, Zhou X, Kar A, Ray P, et al (2011) An ALS-associated mutation affecting TDP-43 enhances protein aggregation, fibril formation and neurotoxicity. Nat Struct Mol Biol 18: 822–830 doi:810.1038/nsmb.2053 21666678 10.1038/nsmb.2053 PMC3357956

[11] Udan-Johns M, Bengoechea R, Bell S, Shao J, Diamond MI, et al. (2013) Prion-like nuclear aggregation of TDP-43 during heat shock is regulated by HSP40/70 chaperones. Human molecular genetics. 10.1093/hmg/ddt408 PMC3857952 23962724

[12] Wang IF, Reddy NM, Shen CK (2002) Higher order arrangement of the eukaryotic nuclear bodies. Proc Natl Acad Sci U S A 99: 13583–13588. Epub 12002 Oct 13582. 10.1073/pnas.212483099 PMC129717 12361981

[13] Zhang YJ, Xu YF, Dickey CA, Buratti E, Baralle F, et al (2007) Progranulin mediates caspase-dependent cleavage of TAR DNA binding protein-43. J Neurosci 27: 10530–10534. 17898224 10.1523/JNEUROSCI.3421-07.2007 PMC6673167

[14] Kabashi E, Lin L, Tradewell ML, Dion PA, Bercier V, et al. (2010) Gain and loss of function of ALS-related mutations of TARDBP (TDP-43) cause motor deficits in vivo. Hum Mol Genet 19: 671–683. Epub 2009 Dec 2003. 10.1093/hmg/ddp534 19959528

[15] Wu LS, Cheng WC, Shen CK (2012) Targeted depletion of TDP-43 expression in the spinal cord motor neurons leads to the development of amyotrophic lateral sclerosis-like phenotypes in mice. J Biol Chem 287: 27335–27344 doi:27310.21074/jbc.M27112.359000. Epub 352012 Jun 359020 22718760 10.1074/jbc.M112.359000 PMC3431639

[16] Hasegawa M, Arai T, Nonaka T, Kametani F, Yoshida M, et al (2008) Phosphorylated TDP-43 in frontotemporal lobar degeneration and amyotrophic lateral sclerosis. Ann Neurol 64: 60–70. 18546284 10.1002/ana.21425 PMC2674108

[17] Tsuji H, Arai T, Kametani F, Nonaka T, Yamashita M, et al (2012) Molecular analysis and biochemical classification of TDP-43 proteinopathy. Brain 135: 3380–3391 doi:3310.1093/brain/aws3230. Epub 2012 Oct 3383 23035040 10.1093/brain/aws230

[18] Arai T, Hasegawa M, Akiyama H, Ikeda K, Nonaka T, et al. (2006) TDP-43 is a component of ubiquitin-positive tau-negative inclusions in frontotemporal lobar degeneration and amyotrophic lateral sclerosis. Biochem Biophys Res Commun 351: 602–611. Epub 2006 Oct 2030. 10.1016/j.bbrc.2006.10.093 17084815

[19] Geser F, Martinez-Lage M, Kwong LK, Lee VM, Trojanowski JQ (2009) Amyotrophic lateral sclerosis, frontotemporal dementia and beyond: the TDP-43 diseases. J Neurol 256: 1205–1214. Epub 2009 Mar 1207. 10.1007/s00415-009-5069-7 PMC2790321 19271105

[20] Higashi S, Iseki E, Yamamoto R, Minegishi M, Hino H, et al (2007) Concurrence of TDP-43, tau and alpha-synuclein pathology in brains of Alzheimer's disease and dementia with Lewy bodies. Brain research 1184: 284–294. 17963732 10.1016/j.brainres.2007.09.048

[21] Thorpe JR, Tang H, Atherton J, Cairns NJ (2008) Fine structural analysis of the neuronal inclusions of frontotemporal lobar degeneration with TDP-43 proteinopathy. J Neural Transm 115: 1661–1671. Epub 2008 Oct 1631. 10.1007/s00702-008-0137-1 PMC2789307 18974920

[22] Lin WL, Dickson DW (2008) Ultrastructural localization of TDP-43 in filamentous neuronal inclusions in various neurodegenerative diseases. Acta Neuropathol 116: 205–213. Epub 2008 Jul 2008. 10.1007/s00401-008-0408-9 PMC2706695 18607609

[23] Brettschneider J, Libon DJ, Toledo JB, Xie SX, McCluskey L, et al. (2012) Microglial activation and TDP-43 pathology correlate with executive dysfunction in amyotrophic lateral sclerosis. Acta Neuropathol 123: 395–407. Epub 2012 Jan 2011. 10.1007/s00401-011-0932-x PMC3595560 22210083

[24] Johnson BS, Snead D, Lee JJ, McCaffery JM, Shorter J, et al (2009) TDP-43 is intrinsically aggregation-prone, and amyotrophic lateral sclerosis-linked mutations accelerate aggregation and increase toxicity. The Journal of biological chemistry 284: 20329–20339. 19465477 10.1074/jbc.M109.010264 PMC2740458

[25] Furukawa Y, Kaneko K, Watanabe S, Yamanaka K, Nukina N (2011) A seeding reaction recapitulates intracellular formation of Sarkosyl-insoluble transactivation response element (TAR) DNA-binding protein-43 inclusions. J Biol Chem 286: 18664–18672.Epub 12011 Mar 18624. 10.1074/jbc.M111.231209 PMC3099683 21454603

[26] Pesiridis GS, Tripathy K, Tanik S, Trojanowski JQ, Lee VM (2011) A “two-hit” hypothesis for inclusion formation by carboxyl-terminal fragments of TDP-43 protein linked to RNA depletion and impaired microtubule-dependent transport. The Journal of biological chemistry 286: 18845–18855. 21454607 10.1074/jbc.M111.231118 PMC3099701

[27] Nonaka T, Masuda-Suzukake M, Arai T, Hasegawa Y, Akatsu H, et al (2013) Prion-like properties of pathological TDP-43 aggregates from diseased brains. Cell reports 4: 124–134. 23831027 10.1016/j.celrep.2013.06.007

[28] Choksi DK, Roy B, Chatterjee S, Yusuff T, Bakhoum MF, et al. (2013) TDP-43 Phosphorylation by casein kinase I{varepsilon} promotes oligomerization and enhances toxicity in vivo. Human molecular genetics. 10.1093/hmg/ddt498 24105464

[29] Nonaka T, Arai T, Buratti E, Baralle FE, Akiyama H, et al. (2009) Phosphorylated and ubiquitinated TDP-43 pathological inclusions in ALS and FTLD-U are recapitulated in SH-SY5Y cells. FEBS Lett 583: 394–400. Epub 2008 Dec 2025. 10.1016/j.febslet.2008.12.031 19111550

[30] Liachko NF, Guthrie CR, Kraemer BC (2010) Phosphorylation promotes neurotoxicity in a Caenorhabditis elegans model of TDP-43 proteinopathy. J Neurosci 30: 16208–16219. 21123567 10.1523/JNEUROSCI.2911-10.2010 PMC3075589

[31] Zhang YJ, Gendron TF, Xu YF, Ko LW, Yen SH, et al (2010) Phosphorylation regulates proteasomal-mediated degradation and solubility of TAR DNA binding protein-43 C-terminal fragments. Mol Neurodegener 5: 33. 20804554 10.1186/1750-1326-5-33 PMC2941488

[32] Sarkar M, Kuret J, Lee G (2008) Two motifs within the tau microtubule-binding domain mediate its association with the hsc70 molecular chaperone. J Neurosci Res 86: 2763–2773. 18500754 10.1002/jnr.21721 PMC4271846

[33] Jinwal UK, O'Leary JC 3rd, Borysov SI, Jones JR, Li Q, et al. (2010) Hsc70 rapidly engages tau after microtubule destabilization. J Biol Chem 285: 16798–16805. Epub 12010 Mar 16722. 10.1074/jbc.M110.113753 PMC2878041 20308058

[34] Dickey CA, Kamal A, Lundgren K, Klosak N, Bailey RM, et al. (2007) The high-affinity HSP90-CHIP complex recognizes and selectively degrades phosphorylated tau client proteins. J Clin Invest 117: 648–658. PMCID: PMC1794119. 10.1172/JCI29715 PMC1794119 17304350

[35] Dickey C, Kraft C, Jinwal U, Koren J, Johnson A, et al. (2009) Aging analysis reveals slowed tau turnover and enhanced stress response in a mouse model of tauopathy. Am J Pathol 174: 228–238.Epub 2008 Dec 2012. 10.2353/ajpath.2009.080764 PMC2631335 19074615

[36] Jinwal UK, Abisambra JF, Zhang J, Dharia S, O'Leary JC, et al. (2012) Cdc37/Hsp90 protein complex disruption triggers an autophagic clearance cascade for TDP-43 protein. J Biol Chem 287: 24814–24820. Epub 22012 Jun 24816. 10.1074/jbc.M112.367268 PMC3397908 22674575

[37] Freibaum BD, Chitta RK, High AA, Taylor JP (2010) Global analysis of TDP-43 interacting proteins reveals strong association with RNA splicing and translation machinery. Journal of proteome research 9: 1104–1120. 20020773 10.1021/pr901076y PMC2897173

[38] Wang JZ, Grundke-Iqbal I, Iqbal K (2007) Kinases and phosphatases and tau sites involved in Alzheimer neurofibrillary degeneration. Eur J Neurosci 25: 59–68. 17241267 10.1111/j.1460-9568.2006.05226.x PMC3191918

[39] Sahara N, Maeda S, Yoshiike Y, Mizoroki T, Yamashita S, et al (2007) Molecular chaperone-mediated tau protein metabolism counteracts the formation of granular tau oligomers in human brain. J Neurosci Res 85: 3098–3108. 17628496 10.1002/jnr.21417

[40] Patterson KR, Ward SM, Combs B, Voss K, Kanaan NM, et al. (2011) Heat shock protein 70 prevents both tau aggregation and the inhibitory effects of preexisting tau aggregates on fast axonal transport. Biochemistry 50: 10300–10310. Epub 12011 Nov 10308. 10.1021/bi2009147 PMC3387688 22039833

[41] Lashuel HA, Hartley D, Petre BM, Walz T, Lansbury PT Jr (2002) Neurodegenerative disease: amyloid pores from pathogenic mutations. Nature 418: 291. 10.1038/418291a 12124613

[42] Johnson BS, Snead D, Lee JJ, McCaffery JM, Shorter J, et al (2009) TDP-43 is intrinsically aggregation-prone and ALS-linked mutations accelerate aggregation and increase toxicity. The Journal of biological chemistry 22: 22. 10.1074/jbc.M109.010264 PMC2740458 19465477

[43] Combs B, Voss K, Gamblin TC (2011) Pseudohyperphosphorylation has differential effects on polymerization and function of tau isoforms. Biochemistry 50: 9446–9456 doi:9410.1021/bi2010569. Epub 2012011 Oct 2010517 21942206 10.1021/bi2010569 PMC3224825

[44] Dou F, Netzer WJ, Tanemura K, Li F, Hartl FU, et al. (2003) Chaperones increase association of tau protein with microtubules. Proc Natl Acad Sci U S A 100: 721–726. Epub 2003 Jan 2009. 10.1073/pnas.242720499 PMC141063 12522269

[45] Sahara N, Maeda S, Yoshiike Y, Mizoroki T, Yamashita S, et al (2007) Molecular chaperone-mediated tau protein metabolism counteracts the formation of granular tau oligomers in human brain. J Neurosci Res 85: 3098–3108. 17628496 10.1002/jnr.21417

[46] Miyata Y, Yahara I (1992) The 90-kDa heat shock protein, HSP90, binds and protects casein kinase II from self-aggregation and enhances its kinase activity. The Journal of biological chemistry 267: 7042–7047. 1551911

[47] Masliah E, Iimoto DS, Mallory M, Albright T, Hansen L, et al (1992) Casein kinase II alteration precedes tau accumulation in tangle formation. The American journal of pathology 140: 263–268. 1739121 PMC1886426

[48] Iimoto DS, Masliah E, DeTeresa R, Terry RD, Saitoh T (1990) Aberrant casein kinase II in Alzheimer's disease. Brain research 507: 273–280. 2337767 10.1016/0006-8993(90)90282-g

[49] Herman AM, Khandelwal PJ, Stanczyk BB, Rebeck GW, Moussa CE (2011) beta-amyloid triggers ALS-associated TDP-43 pathology in AD models. Brain research 1386: 191–199. 21376022 10.1016/j.brainres.2011.02.052 PMC3073036

[50] Li HY, Yeh PA, Chiu HC, Tang CY, Tu BP (2011) Hyperphosphorylation as a defense mechanism to reduce TDP-43 aggregation. PloS one 6: e23075. 21850253 10.1371/journal.pone.0023075 PMC3151276

[51] Brady OA, Meng P, Zheng Y, Mao Y, Hu F (2011) Regulation of TDP-43 aggregation by phosphorylation and p62/SQSTM1. Journal of neurochemistry 116: 248–259. 21062285 10.1111/j.1471-4159.2010.07098.x

[52] Zhao M, Ma J, Zhu HY, Zhang XH, Du ZY, et al (2011) Apigenin inhibits proliferation and induces apoptosis in human multiple myeloma cells through targeting the trinity of CK2, Cdc37 and Hsp90. Molecular cancer 10: 104. 21871133 10.1186/1476-4598-10-104 PMC3170639

[53] Neumann M, Igaz LM, Kwong LK, Nakashima-Yasuda H, Kolb SJ, et al (2007) Absence of heterogeneous nuclear ribonucleoproteins and survival motor neuron protein in TDP-43 positive inclusions in frontotemporal lobar degeneration. Acta neuropathologica 113: 543–548. 17415574 10.1007/s00401-007-0221-x

[54] Johnson BS, Snead D, Lee JJ, McCaffery JM, Shorter J, et al. (2009) TDP-43 is intrinsically aggregation-prone, and amyotrophic lateral sclerosis-linked mutations accelerate aggregation and increase toxicity. J Biol Chem 284: 20329–20339. Epub 22009 May 20322. 10.1074/jbc.M109.010264 PMC2740458 19465477

[55] Hernandez MP, Sullivan WP, Toft DO (2002) The assembly and intermolecular properties of the hsp70-Hop-hsp90 molecular chaperone complex. J Biol Chem 277: 38294–38304. Epub 32002 Aug 38292. 10.1074/jbc.M206566200 12161444

[56] Lin WL, Dickson DW (2008) Ultrastructural localization of TDP-43 in filamentous neuronal inclusions in various neurodegenerative diseases. Acta neuropathologica 116: 205–213. 18607609 10.1007/s00401-008-0408-9 PMC2706695

[57] Schneider CA, Rasband WS, Eliceiri KW (2012) NIH Image to ImageJ: 25 years of image analysis. Nat Methods 9: 671–675. 22930834 10.1038/nmeth.2089 PMC5554542

