# Correction: Casein Kinase II Induced Polymerization of Soluble TDP-43 into Filaments Is Inhibited by Heat Shock Proteins

**DOI:** 10.1371/journal.pone.0100026

**Published:** 2014-06-06

**Authors:** The PLOS ONE Staff

There is an error in the spelling of the affiliation for all of the authors. All authors are affiliated with: Department of Neuroscience, Mayo Clinic, Jacksonville, Florida, United States of America.
